# M6A-mediated-upregulation of lncRNA BLACAT3 promotes bladder cancer angiogenesis and hematogenous metastasis through YBX3 nuclear shuttling and enhancing NCF2 transcription

**DOI:** 10.1038/s41388-023-02814-3

**Published:** 2023-08-23

**Authors:** Jinbo Xie, Hui Zhang, Keyi Wang, Jinliang Ni, Xiaoying Ma, Christopher J. Khoury, Viktor Prifti, Brock Hoard, Eric G. Cerenzia, Lei Yin, Houliang Zhang, Ruiliang Wang, Dong Zhuo, Weipu Mao, Bo Peng

**Affiliations:** 1 Department of Urology, Shanghai Tenth People’s Hospital, School of Medicine, Tongji University, Shanghai, 200072 China; 2 Department of Urology, The First Affiliated Hospital of Wannan Medical College (Yijishan Hospital of Wannan Medical College), Wuhu, 241001 China; 3 Department of Laboratory Medicine and Pathobiology, University of Toronto, Toronto, ON M5S 1A1 Canada; 4 Department of Laboratory Medicine, LKSKI-Keenan Research Centre for Biomedical Science, St. Michael’s Hospital, Toronto, ON M5B 1W8 Canada; 5 Department of Anesthesiology and Perioperative Medicine, Shanghai Fourth People’s Hospital, School of Medicine, Tongji University, Shanghai, 200434 China; 6 Department of Urology, Ruijin Hospital, Shanghai Jiaotong University School of Medicine, Shanghai, 200025 China; 7 Department of Urology, Affiliated Zhongda Hospital of Southeast University, Nanjing, 210009 China

**Keywords:** Bladder cancer, Prognostic markers

## Abstract

Lymphatic metastasis is recognized as the leading manner of metastasis in bladder cancer (BLCa), but hematogenous metastasis accounts for a majority of cancer-associated deaths. The past two decades have witnessed tremendous attention in long non-coding RNAs (lncRNAs), which are a new hope for the development of targeted drug therapy for metastatic cancers; however, the underlying mechanism of lncRNAs involved in BLCa hematogenous metastasis remains to be elucidated. Here, we identified BLCa-associated transcript 3 (BLACAT3), a lncRNA, which was aberrantly upregulated in BLCa and corelated with poor prognosis of patients with muscle-invasive bladder cancer. Methodologically, m6A epitranscriptomic microarray, RNA sequencing and mass spectrometry (MS) were used to screen the key molecules of the regulatory axis. Functional assays, animal models and clinical samples were used to explore the roles of BLACAT3 in BLCa in vitro and in vivo. Mechanistically, m6A modification contributes to BLACAT3 upregulation by stabilizing RNA structure. BLACAT3 recruits YBX3 to shuttle into the nucleus, synergistically enhances NCF2 transcription, and promotes BLCa angiogenesis and hematogenous metastasis by activating downstream NF-κB signaling. Our findings will develop prognosis prediction tools for BLCa patients and discover novel therapeutic biological targets for metastatic BLCa.

## Introduction

Bladder cancer (BLCa) is the second most common genitourinary malignancy with approximately 549,000 new confirmed cases and 200,000 deaths worldwide each year [1]. Patients with non-muscle-invasive BLCa (NMIBC) undergo bladder-sparing surgeries and standardized intravesical therapy, yet there is a 30–45% chance of converting to muscle-invasive BLCa (MIBC) within 5 years, some even directly progressing to metastatic BLCa [2]. About 50% of MIBC patients may suffer local or distant recurrence after radical cystectomy (RC) [3]. As for metastatic BLCa, platinum-based chemotherapy is currently the first-line therapeutic, but the overall response rate is unfortunately less than 50% [4]. While alternative options such as radiotherapy and checkpoint inhibitors are available, both carry their own major drawbacks and new target drugs are still in clinical trial development [5–9]. Therefore, understanding the mechanism of BLCa metastasis is essential so that novel therapeutic targets can be developed to improve patient outcomes.

Previous studies have primarily focused on the mechanism of lymphangiogenesis and lymphatic metastasis in BLCa, because lymphatic metastasis is considered the leading metastatic manner in BLCa [10–13]. In more severe cases, BLCa cells metastasize to distant organs such as liver, lung, and bone through the hematogenous approach which is more associated with cancer-associated mortality [14–17] but remains poorly studied. Angiogenesis refers to the forming of new blood vessels, which can provide the necessary blood supply for invasive tumor growth and metastasis [18]. Accumulating evidence show reactive oxygen species (ROS) catalyzed by intracellular NADPH oxidase participate in angiogenesis, invasion and metastasis of BLCa by activating downstream signaling [19, 20]. NCF2/p67phox is one of the two important activating subunits of NADPH oxidase [21, 22], and plays an irreplaceable role in the biological function of NADPH oxidase. NCF2/p67phox expression has been reported to be upregulated in clear cell renal cell carcinoma (ccRCC) [23], urothelial carcinoma [20], malignant glioma [24, 25], hepatocellular carcinoma [26, 27] and colorectal carcinoma [28], and associated with poor prognosis. However, the regulatory mechanism of NCF2/p67phox in BLCa angiogenesis and metastasis remains unknown.

LncRNAs are a class of transcripts that contain 200–100,000 nucleotides and cannot be translated into peptides [29, 30]. LncRNAs have garnered significant attention over the past two decades as potential targets for metastatic BLCa drug therapy; however, the mechanism by which lncRNAs are involved in BLCa hematogenous metastasis remains to be elucidated. Using BLCa specimens and patient data, and with the help of m6A epitranscriptomic microarray technology and bioinformatics analysis based on public high-throughput data, we identified a potential target lncRNA, LINC01535–204, which we named BLCa-associated transcript 3 (BLACAT3). Our data showed BLACAT3 was highly expressed in BLCa tissues and positively correlated with poor prognosis in MIBC patients. Furthermore, we analyzed the regulatory mechanism of BLACAT3 on the malignant behavior of BLCa cells and the prognosis of the disease from the molecular, cellular, mammalian, and clinical levels. Using several m6A-associated assays, we demonstrated that m6A modification contributes to BLACAT3 upregulation by stabilizing RNA structure. Our data confirmed BLACAT3 regulates the expression of NCF2/p67phox by interacting with RNA binding protein (RBP) YBX3 to mediate BLCa angiogenesis and hematogenous metastasis. Overall, this study has provided strong insights and an experimental basis for the identification of novel therapeutic targets for metastatic BLCa.

## Results

### BLACAT3 exhibits high-abundance m6A modification and elevated expression in MIBC tissues and is a potential prognostic indicator

**Fig. 1:**
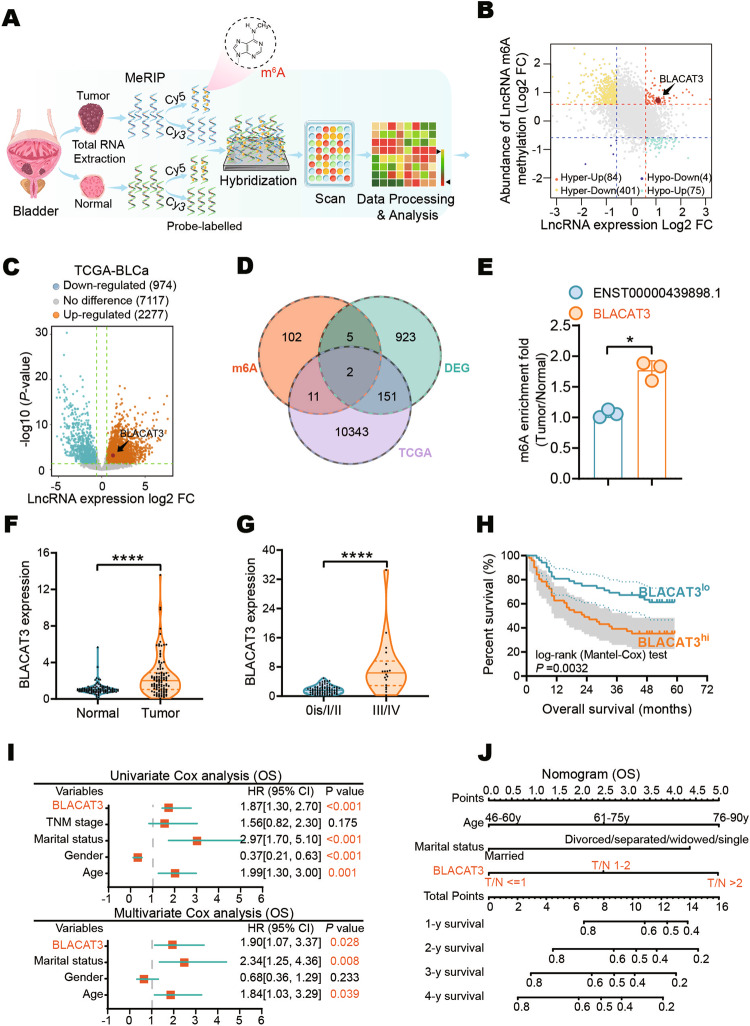
BLACAT3 identification and its relationship with BLCa patient prognosis. **A** Flowchart of m6A epitranscriptomic microarray. **B** Differentially m6A modified lncRNAs (abs(log_2_FC) > 0.585, *P* < 0.05) and differentially expressed lncRNAs ((abs(log_2_FC) > 0.585) between paired BLCa and adjacent normal tissues (n = 3). **C** Volcano plot of differential lncRNA expression profile between 411 BLCa tissues and 19 normal bladder tissues based on TCGA data (abs(log_2_FC) > 0.585, *P* < 0.05). **D** Venn diagrams of microarray-derived differential lncRNA m6A modification and expression profiles intersected with lncRNA expression profiles from the TCGA dataset. **E** MeRIP and qRT-PCR were conducted to detect m6A modification levels of BLACAT3 and ENST00000439898.1 (*n* = 3). **F** qRT-PCR was used to detect BLACAT3 expression between paired BLCa and adjacent normal tissues (*n* = 104). **G** Comparison of BLACAT3 expression between earlier stage (*n* = 86) and advanced stage (n = 18). **H** Kaplan-Meier analysis of the relationship between BLACAT3 expression and OS of BLCa patients (Log-rank (Mantel-Cox) test, *P* < 0.001). 104 BLCa patients were divided into high and low expression groups based on the median value of relative BLACAT3 expression. **I** Univariate and multivariate regression analyses were conducted to screen the independent predictors associated with OS in BLCa patients. Hazard ratio (HR) and corresponding 95% confidence intervals (CI) are shown. **J** Nomogram was constructed to predict the prognosis of BLCa patients undergoing RC. Statistical significance was assessed with a two-tailed Student’s t test between two groups, *P < 0.05, **** *P* < 0.0001.

To identify novel potential vulnerabilities associated with BLCa progression, we screened the differential m6A modification and expression level profile of lncRNAs between three pairs of fresh tumor tissues and adjacent normal tissues by human m6A epitranscriptomic microarray (Fig. 1A, sFig. 1A, B, Supplementary Tables 7, 8). We then focused on 84 lncRNAs with co-upregulation of m6A modification and expression level (Fig. 1B). We further extracted the BLCa sequencing dataset from The Cancer Genome Atlas (TCGA) (Fig. 1C). Two lncRNA candidates, LINC01535-204 and ENST00000439898.1 (Fig. 1D, Supplementary Table 9), were identified from the intersection of the three datasets. M6A RNA immunoprecipitation (MeRIP) and quantitative real-time PCR (qRT-PCR) analyses verified that, the relative m6A modification of BLACAT3 in BLCa, compared with paired normal tissues, was significantly higher than that of ENST00000439898.1 (Fig. 1E). BLACAT3 is 815nt long and is located in Chromosome 19: 37,251,922-37,265,473 (sFig. 1C). GSEA based on microarray data suggested that BLACAT3 is closely involved in biological functions including cell division, protein binding, HIF-1 signaling pathway, and tumor metabolism (sFig. 1D-G). We also confirmed that BLACAT3 does not have the ability to encode a protein by Coding Potential Calculator 2 [31] (sFig. 1H, I).

We detected the BLACAT3 expression in 104 pairs of BLCa and adjacent normal tissues using qRT-PCR. It was found that BLACAT3 expression in BLCa tissue was significantly higher than in normal tissues (Fig. 1F; sFig. 1J). Moreover, BLACAT3 expression was significantly higher in TNM stage III/IV BLCa compared to 0is/I/II BLCa (Fig. 1G). Additionally, MIBC patients with higher BLACAT3 expression had worse overall survival (OS) and disease-specific survival (DSS) (Fig. 1H; sFig. 1K). We used univariate and multivariate regression analyses to screen out three key variables including BLACAT3 expression, marital status, and gender (Fig. 1I) and established a prognostic risk prediction model for MIBC patients who underwent RC (Fig. 1J). Receiver operating characteristic curves and decision curve analysis demonstrated the high prediction accuracy and fitting degree of this model (sFig. 1L, M).

### M6A modification mediates BLACAT3 upregulation by enhancing RNA stability, and promotes malignant behaviors of BLCa cells

**Fig. 2:**
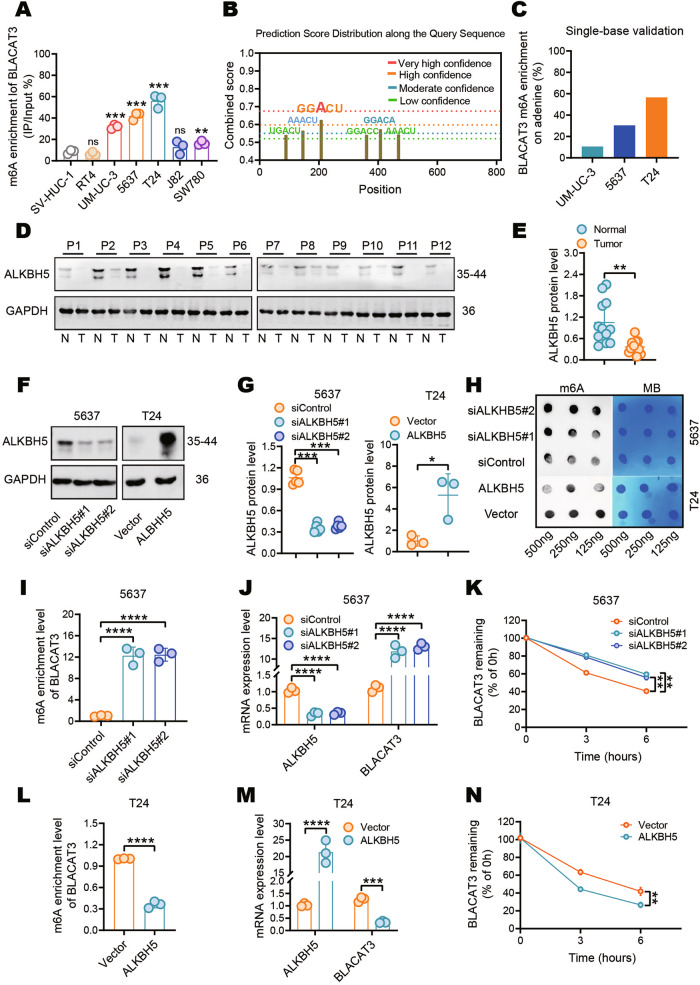
Effect of m6A modification on BLACAT3 expression in BLCa. **A** MeRIP and qRT-PCR were used to detect the m6A modification levels of BLACAT3 in different BLCa cell lines (RT4, UM-UC-3, 5637, T24, J82 and SW780) and the normal urothelial cell line (SV-HUC-1). **B** The SRAMP prediction server (https://www.cuilab.cn/sramp) was used to predict potential m6A modification motifs in the BLACAT3 sequence. **C** Single-base mapping of m6A against adenine in the “GGACU” motif was performed to detect the m6A abundance of BLACAT3 in several BLCa cell lines (UM-UC-3, 5637 and T24). **D**, **E** WB were performed to detect the protein level of ALKBH5 in paired MIBC and adjacent normal tissues (*n* = 12). **F**, **G** WB were performed to verify the knockdown efficiency of ALKBH5 in 5637 cells and the overexpression efficiency of ALKBH5 in T24 cells. **H** Anti-m6A dot blot assay detected the effect of ALKBH5 knockdown or overexpression on the overall m6A modification level of 5637 or T24 cells. **I** MeRIP and qRT-PCR detected the effect of ALKBH5 knockdown on BLACAT3 m6A enrichment in 5637 cells. **J** QRT-PCR quantified ALKBH5 knockdown efficiency and BLACAT3 expression in 5637 cells. **K** QRT-PCR examined the effect of ALKBH5 knockdown on the half-life of BLACAT3 in 5637 cells pretreated with actinomycin-D (5 μg/mL). **L** MeRIP and qRT-PCR assays were conducted to detect the effect of ALKBH5 overexpression on BLACAT3 m6A enrichment in T24 cells. **M** QRT-PCR assay was performed to detect the overexpression efficiency of ALKBH5 and BLACAT3 expression in T24 cells. **N** QRT-PCR assay was performed to examine the effect of ALKBH5 overexpression on the half-life of BLACAT3 in T24 cells pretreated with actinomycin-D (5 μg/mL). ns no significance. Statistical significance was assessed using two-tailed Student’s *t* test between two groups, *P < 0.05, **P < 0.01, ***P < 0.001, ****P < 0.0001.

MeRIP and qRT-PCR analyses verified BLACAT3 m6A modification and expression level were consistently higher than those of SV-HUC-1 in most BLCa cell lines, with the most significant differences in T24, 5637, and UM-UC-3 (Fig. 2A, sFig. 2A). Dot blot assay showed that total m6A modification levels of BLACAT3 in the three cell lines were higher than in SV-HUC-1 (sFig. 2B). SRAMP prediction server [32] identified 6 potential m6A modification motifs in the BLACAT3 sequence (Fig. 2B). We then focused on the GGACU motif, which exhibits the highest prediction confidence. Single-base mapping of m6A [33] showed that the m6A modification ratios of the GGACU motif in UM-UC-3, 5637 and T24 cell lines were 10.63%, 30.41% and 56.63%, respectively (Fig. 2C). Therefore, we verified the high m6A modification ratio of the BLACAT3 sequence in BLCa cells.

METTL3, METTL14, WTAP, FTO and ALKBH5 are five well-known m6A modification related enzymes, of which the first three are methyltransferases and the last two are demethylases [34]. Using small interference RNAs (siRNAs) (sFig. 2C), we found that silencing ALKBH5 most significantly upregulated the m6A modification level of BLACAT3 (sFig. 2D). Western blots (WB) demonstrated that ALKBH5 protein level in BLCa tissue was significantly lower than that of matched normal tissue (Fig. 2D, E), and lower in BLCa cell lines compared to normal urothelial cell line (sFig. 2E).

To investigate the effect of m6A modification on BLACAT3 expression, we overexpressed or silenced ALKBH5 in 5637 and T24 cells respectively (Fig. 2F, G). Dot blot assay showed that the overall m6A abundance of BLCa cells was significantly up- or down-regulated after ALKBH5 knockdown or overexpression (Fig. 2H), respectively. MeRIP and qRT-PCR analyses demonstrated that ALKBH5 knockdown significantly upregulated both m6A modification and expression of BLACAT3 in 5637 cells (Fig. 2I, J). Actinomycin D is an inhibitor of RNA transcription and is commonly used to explore RNA half-life [35, 36]. We used qRT-PCR to quantify residual BLACAT3 at different time points and found that silencing ALKBH5 could significantly reduce the degradation rate of BLACAT3 at 3 and 6 h after the addition of actinomycin D (Fig. 2K). ALKBH5 overexpression in T24 cells could significantly downregulate m6A modification levels and BLACAT3 expression (Fig. 2L, M), and significantly shorten the RNA half-life of BLACAT3 (Fig. 2N).

We also explored the effect of m6A modification on the malignant behaviors of BLCa cells. CCK-8 assay showed that up- or down-regulation of m6A modification could promote or inhibit the BLCa cell proliferation (sFig. 3A, B), respectively. Human umbilical vein endothelial cell (HUVEC) tube formation and transwell migration assays demonstrated that up- or down-regulation of m6A modification could respectively enhance or inhibit the angiogenesis and migration of BLCa cells (sFig. 3C–H).

### BLACAT3 Upregulation promotes proliferation of BLCa cell both in vitro and in vivo

**Fig. 3:**
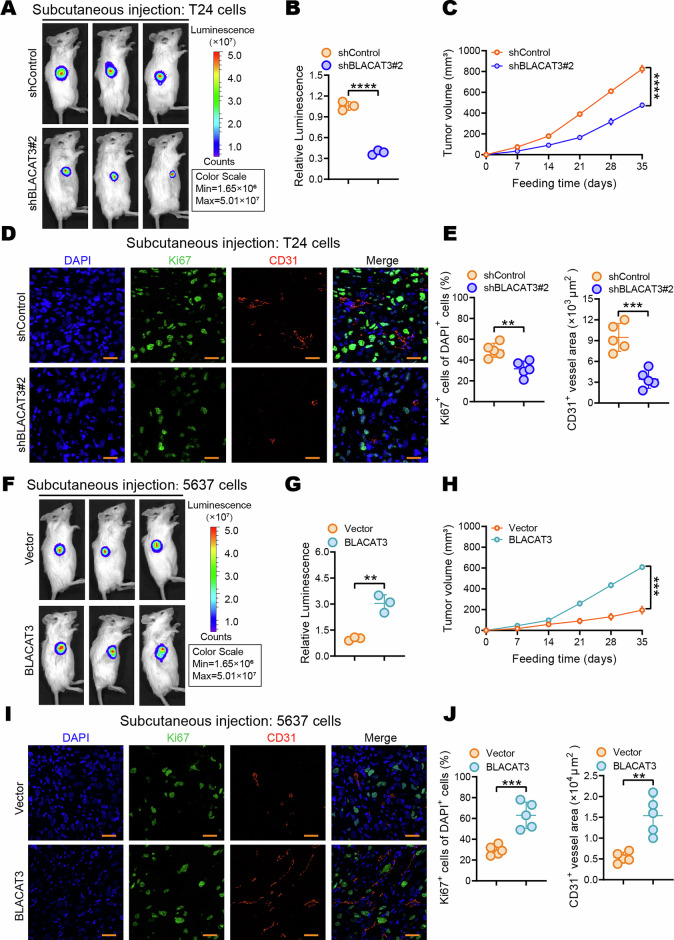
Effects of BLACAT3 on BLCa proliferation in vivo. **A**, **B** In vivo imaging was performed on day 28 after subcutaneous injection of BLACAT3 stable knockdown T24 cells into NSG mice, fluorescent quantitative statistics were performed on representative images. **C** Tumor volume was measured every 7 days after subcutaneous injection. **D**, **E** Anti-Ki67 and anti-CD31 IF staining was conducted to explore the effect of BLACAT3 knockdown on the proliferation and angiogenesis of T24 cells in vivo. Image J software was used for semi-quantitative analysis of representative immunofluorescent images. Scale bars: 20 μm. **F**, **G** In vivo imaging was performed on day 28 after subcutaneous injection of 5637 cells with stable BLACAT3 overexpression into NSG mice, and fluorescence quantitative statistics were performed on representative images. **H** Tumor volume was measured every 7 days after subcutaneous inoculation of 5637 cells. **I**, **J** Anti-Ki67 and anti-CD31 immunofluorescent staining were performed to assess the effects of BLACAT3 overexpression on the proliferation and angiogenesis of 5637 cells in vivo. Image J software was used to perform semi-quantitative analyzing of representative fluorescent images. Scale bars: 20 μm. Statistical significance was assessed using two-tailed Student’s *t* test between two groups, **P < 0.01, ***P < 0.001, ****P < 0.0001.

To explore the effect of BLACAT3 on the malignant behaviors of BLCa cells, we constructed the stable T24 strain with BLACAT3 knockdown and 5637 strain with BLACAT3 overexpression for establishing the subcutaneous xenograft model (sFig. 4A, B, sFig. 5A, B). Both cell stains also stably expressed the luciferase gene. On the 28th day after subcutaneous injection of BLCa cells, in vivo imaging showed that BLACAT3 knockdown and overexpression respectively inhibited and promoted tumor growth (Fig. 3A, B, F, G). We plotted tumor growth curves of subcutaneous tumors (Fig. 3C, H). At the endpoint, gross samples of the subcutaneous tumors were isolated and photographed (sFigs. 4Cs, 5C). Tumor weights were recorded (sFigs. 4D, 5D), and the tumor pathology was confirmed by haematoxylin and eosin (H&E) staining (sFigs. 4E, 5E). Ki67 and CD31 are commonly used to evaluate proliferation and tumor angiogenesis [37]. Immunofluorescence (IF) staining showed that knockdown or overexpression of BLACAT3 could significantly inhibit or promote BLCa cell proliferation and angiogenesis in vivo (Fig. 3D, E, I, J). The 5-Ethynyl-2’-deoxyuridine (EdU) (sFigs. 4F-H, 5F-H) and plate cloning assays (sFigs. 4I-K, 5I-K) showed that the effects of BLACAT3 on BLCa cell proliferation was consistent with our findings in vivo.

### BLACAT3 promotes angiogenesis and hematogenous metastasis in BLCa

**Fig. 4:**
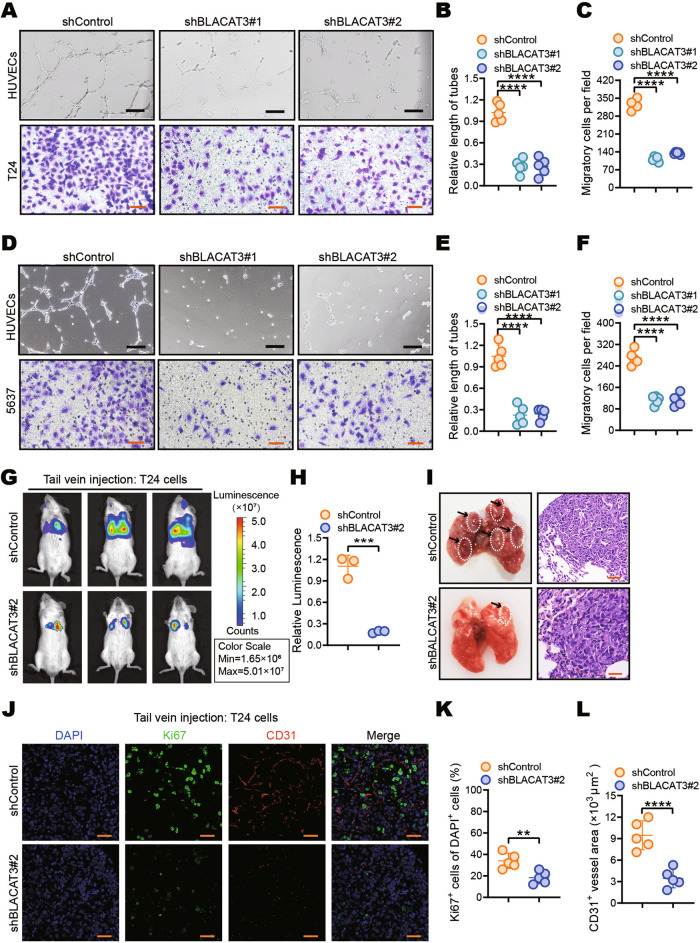
Effects of BLACAT3 on BLCa angiogenesis and migration in vitro and in vivo. HUVECs tube formation and transwell migration assay were performed to detect the effects of BLACAT3 knockdown on tumor angiogenesis and migration in T24 (**A**–**C**) and 5637 cells (**D**–**F**). Scale bars: 2 mm (black lines), 500 μm (orange lines). **G**, **H** In vivo imaging was performed on day 28 after tail vein injection of T24 cells with stable BLACAT3 knockdown into NSG mice, and fluorescent quantitative statistics were performed on representative images. **I** NSG mice were sacrificed on the 28th day after the lung metastasis model was constructed, and the gross lung samples were isolated, fixed, embedded and sliced, and then H&E staining was performed. Scale bars: 30 μm. **J–L** Anti-Ki67 and anti-CD31 immunofluorescent staining were performed to assess the T24 cell proliferation and angiogenesis in vivo. and fluorescent quantitative statistics were performed on representative pictures. Scale bars: 20 μm. Statistical significance was assessed using two-tailed Student’s *t* test between two groups, **P < 0.01, ***P < 0.001, ****P < 0.0001.

HUVEC tube formation assay showed that the culture supernatant of BLCa cells with BLACAT3 knockdown could inhibit tube formation (Fig. 4A, B, D, E), while BLACAT3 overexpression could promote tube formation (sFig. 7A, B, E, F). Transwell migration and scratch healing assays consistently showed that BLACAT3 knockdown significantly inhibited the migration of T24 and 5637 cells (Fig. 4A, C, D, F, sFig. 6), while BLACAT3 overexpression significantly promoted the migration of T24 and 5637 cells (sFig. 7A, C, D, E, G, H).

We then used the T24 strain with BLACAT3 knockdown and 5637 strain with BLACAT3 overexpression to establish the hematogenous metastasis models. Both cell strains also stably expressed the luciferase gene. In vivo imaging on the 28th day after tail vein injection (Fig. 4G, H) and the H&E staining results of gross lung tissue samples at the 35th day of the study (Fig. 4I) consistently showed that BLACAT3 knockdown could significantly inhibit pulmonary metastasis. Conversely, in vivo imaging (sFig. 8A, B) and H&E staining (sFig. 8D) demonstrated that BLACAT3 overexpression significantly promoted BLCa pulmonary metastasis. Interestingly, BLCa metastases were exclusively observed in the kidneys of mice overexpressing BLACAT3 (sFig. 8C). Next, we performed IF staining using anti-Ki67 and anti-CD31 antibodies. It was showed that BLACAT3 knockdown significantly inhibited the proliferation and angiogenesis of pulmonary metastatic cancer cells in vivo (Fig. 4J–L).

### BLACAT3 regulates NCF2 expression

**Fig. 5:**
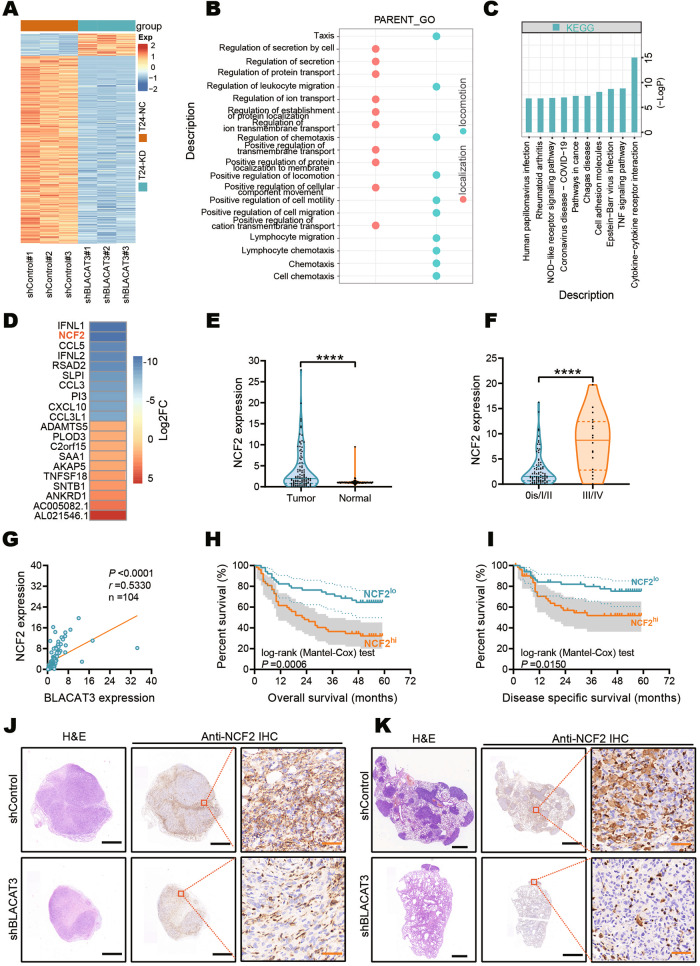
NCF2 identification and its interaction with BLACAT3. **A** Heatmap showed differentially expressed mRNAs in T24 cells with BLACAT3 knockdown. Abs(log_2_(FC)) > 0.585 and *P* < 0.05 were set as the thresholds. **B** Gene Ontology (GO) analysis showed that BLACAT3 knockdown was involved in biological functions related to movement and localization. **C** KEGG enrichment analysis revealed the top 10 signaling pathway regulated by BLACAT3. **D** Heatmap showed top10 mRNAs down- and up-regulated by BLACAT3 knockdown. **E** QRT-PCR detected the NCF2 expression between paired BLCa and adjacent normal tissues (*n* = 104). **F** Relative expression (Tumor/Normal ratio) of NCF2 between earlier TNM stage group (*n* = 86) and advanced stage group (*n* = 18). **G** Correlation analysis between BLACAT3 and NCF2 expression exhibited a positive correlation, *P* < 0.0001. **H, I** Kaplan-Meier survival curves showed the effect of NCF2 expression on the OS of BLCa patients (Log-rank (Mantel-Cox) test, *P* < 0.001) and DSS of BLCa patients (Log-rank (Mantel-Cox) test, *P* < 0.05). A total of 104 BLCa patients were divided into high expression group and low expression group by the median of relative NCF2 expression. **J** NSG mice were subcutaneously injected using T24 cells with BLACAT3 stable knockdown, and the mice were sacrificed on the 28th day. The subcutaneous tumor was fixed, embedded in sections, and then H&E staining and anti-NCF2 IHC staining were performed. **K** The mice were sacrificed on the 28th day after tail vein injection, and the gross lung samples were isolated, fixed and embedded, and then H&E staining and anti-NCF2 IHC staining were conducted. Scale bars: 1.6 mm (black lines), 40 μm (orange lines). Statistical significance was assessed using two-tailed Student’s *t* test between two groups, ****P < 0.0001.

Using transcriptome sequencing in T24 cells with BLACAT3 knockdown, we screened 49 upregulated and 412 downregulated mRNAs (Fig. 5A and sFig. 9A, abs (log_2_(FC)) > 0.585, *P* < 0.05). GO analysis showed that BLACAT3 was involved in the regulation of multiple biological processes related to cell movement and localization (Fig. 5B). Kyoto Encyclopedia of Genes and Genomes (KEGG) pathway enrichment analysis showed that BLACAT3 knockdown could activate several cancer-related signaling pathways including the TNF/NF-κB signaling pathway (Fig. 5C). Furthermore, we summarized the biological function entries and Wikipathways results (sFig. 9B, D). Transcriptome sequencing data showed that NCF2 was the second most downregulated gene after BLACAT3 knockdown (Fig. 5D). Therefore, we hypothesized that NCF2 expression could be regulated by BLACAT3.

We verified that NCF2 was significantly upregulated in BLCa tumor tissues by qRT-PCR analysis (Fig. 5E and sFig. 9C). NCF2 expression in advanced BLCa tissues was higher than that in early stage BLCa tissues (Fig. 5F). Interestingly, the mRNA levels of BLACAT3 and NCF2 were significantly positively correlated (Fig. 5G). We analyzed the association of NCF2 levels with BLCa patient prognosis and found that OS and DSS of patients with higher NCF2 expression was significantly poorer than patients with relatively lower NCF2 expression (Fig. 5H, I). In addition, we validated the regulatory relationship between BLACAT3 and NCF2 in mouse-derived samples. Immunohistochemical (IHC) staining demonstrated that BLACAT3 knockdown suppressed NCF2 protein level in both subcutaneous and metastatic foci (Fig. 5J, K).

### Knockdown of NCF2 inhibits angiogenesis and migration of BLCa cells, and exhibits the synergistic effect with BLACAT3 knockdown

**Fig. 6:**
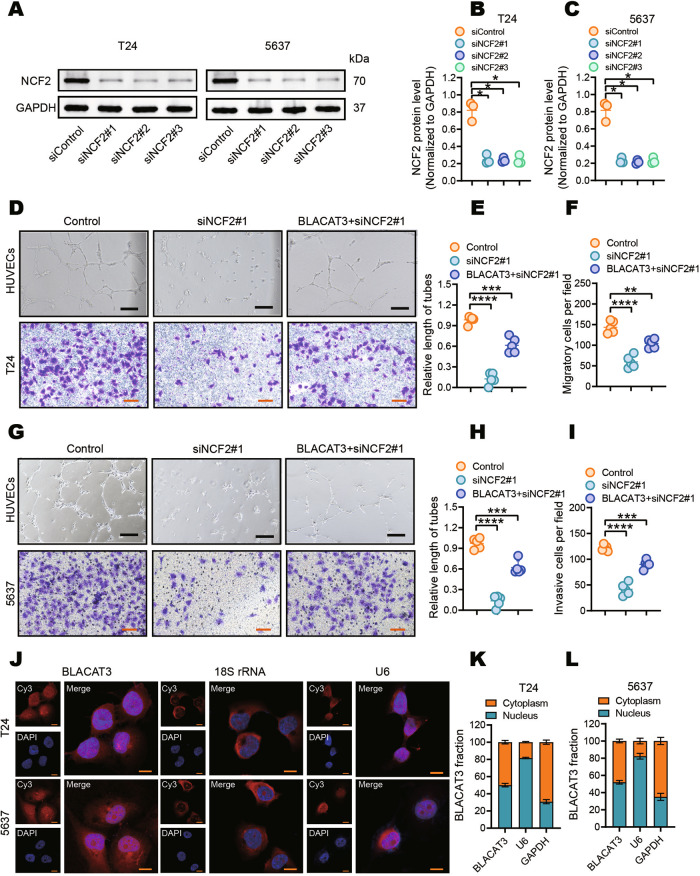
Effects of NCF2 on BLCa angiogenesis and migration in vitro. **A–C** WB verified NCF2 knockdown efficiency in T24 and 5637 cells. **D–I** HUVEC tube formation and transwell migration assays were used to investigate the effects of NCF2 knockdown on pro-angiogenesis and migratory abilities of T24 cells (**D–F**) and 5637 cells (**G–I**). Scale bars: 2 mm (black lines), 500 μm (orange lines). **J** Fluorescence in situ hybridization (FISH) assay detected the subcellular distribution of BLACAT3 in T24 and 5637 cells. 18 S rRNA and U6 were respectively used as cytoplasmic and nuclear internal reference biomarkers. Scale bars: 20 μm. **K**, **L** The subcellular distribution of BLACAT3 in T24 and 5637 cells was detected by qRT-PCR assay after isolation of nuclear and cytoplasmic RNAs. GAPDH and U6 were respectively used as cytoplasmic and nuclear internal reference biomarkers. Statistical significance was assessed using two-tailed Student’s *t* test between two groups, *P < 0.05, **P < 0.01, ***P < 0.001, ****P < 0.0001.

To further explore the oncogenic effect of NCF2 on BLCa, we silenced the NCF2 in T24 and 5637 cells using siRNAs (Fig. 6A–C). Notably, silencing NCF2 partially attenuated the effect of BLACAT3 overexpression on HUVECs tube formation potential (Fig. 6D, E, G, H). Similarly, transwell migration assay indicated that silencing NCF2 significantly inhibited the migration of T24 and 5637 cells, and attenuated the effect of BLACAT3 overexpression on cell migration (Fig. 6D, F, G, I).

RNA FISH (Fig. 6J) and subcellular fractionation-based qRT-PCR analysis (Fig. 6K, L) showed that BLACAT3 was distributed to both the nucleus and cytoplasm of BLCa cells. LncRNAs may interact with chromosomes in the nucleus, miRNA in the cytoplasm, and with protein in both the nucleus and cytoplasm [38]. Thus, we hypothesized that BLACAT3 regulates the expression of NCF2 at the transcriptional or post-transcriptional level by associating with RBPs.

### BLACAT3 binds and induces YBX3 to shuttle into the nucleus

**Fig. 7:**
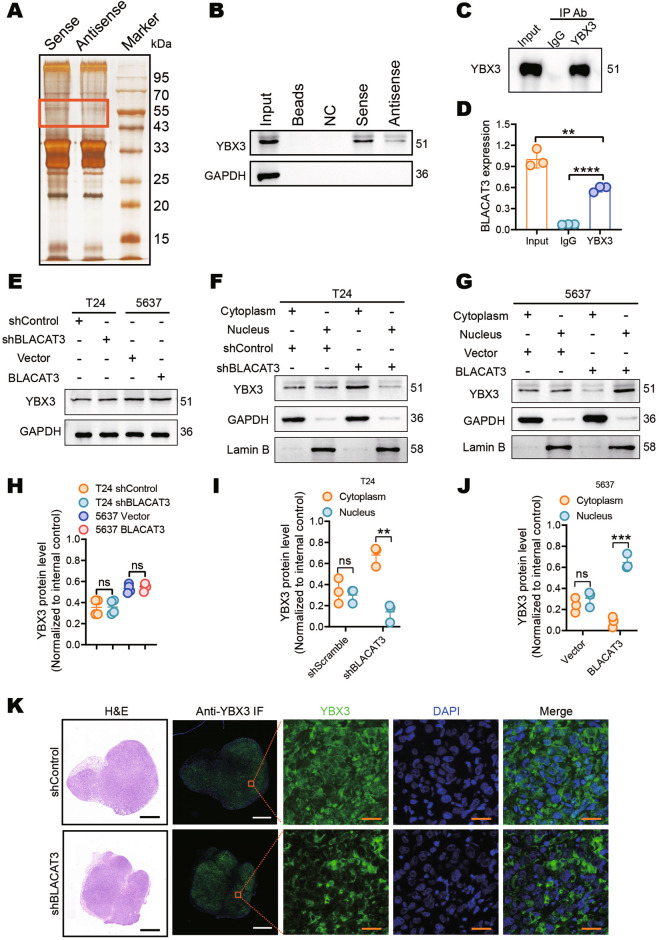
BLACAT3 binds and induces YBX3 to shuttle into the nucleus. **A** RNA pull-down and silver staining showed the molecular weight of BLACAT3 binding proteins. **B** WB assay using the protein sample pulled by BLACAT3 proved that YBX3 is the binding protein of BLACAT3. **C**, **D** RNA Immunoprecipitation (RIP) and qRT-PCR assay confirmed that BLACAT3 can bind with YBX3 protein. **E**, **H** Western blot demonstrated that BLACAT3 knockdown had no effect on YBX3 protein levels in T24 and 5637 cells. **F**, **I** The effect of BLACAT3 knockdown on the subcellular distribution of YBX3 in T24 cells was detected by WB assay after separation of nuclear and cytoplasmic proteins. **G, J** The effect of BLACAT3 overexpression on YBX3 subcellular distribution in 5637 cells was detected by WB assay after separation of nuclear and cytoplasmic proteins. **K** NSG mice were subcutaneously injected with T24 cells with BLACAT3 stable knockdown, and the mice were sacrificed on the 28th day. The subcutaneous tumor was isolated, fixed and embedded, and then H&E staining and anti-YBX3 immunofluorescent (IF) staining assessed the effect of BLACAT3 knockdown on the subcellular distribution of YBX3. Scale bars: 1 mm (black and white lines), 20 μm (orange lines). ns no significance. Statistical significance was assessed using two-tailed Student’s *t* test between two groups, *P < 0.05, **P < 0.01, ***P < 0.001, ****P < 0.0001.

To identify RBPs interacting with BLACAT3, we performed RNA pull-down (Fig. 7A) and MS. We performed GO analysis on 266 proteins identified by MS (Supplementary Table 11) and highlighted proteins enriched for biological functions such as localization, biological process, and signal regulation (sFig. 10A, B). We then performed the pathway enrichment analysis and highlighted some signaling pathways related to protein export and protein processing in the endoplasmic reticulum (sFig. 10C). Wikipathways analysis were enriched for important signaling pathways related to tumor biology, including the VEGFA-VEGFR2 signaling pathway, and mRNA processing (sFig. 10C). We explored 15 potential proteins based on binding affinities to BLACAT3 and focused on YBX3, which is both an RBP and DNA-binding protein (DBP) (sFig. 10D–F). We hypothesized that YBX3 plays a bridge-like role in regulatory mechanism of BLACAT3 on NCF2. We then performed WB and RIP which further demonstrated that BLACAT3 directly binds to YBX3 (Fig. 7B–D).

To better investigate the interaction between BLACAT3 and YBX3, we detected the protein level of YBX3 in T24 and 5637 and found no significant difference in YBX3 protein levels in both BLCa cells with BLACAT3 knockdown or overexpression, suggesting that BLACAT3 had no effect on YBX3 expression (Fig. 7E, H). We then performed nucleocytoplasmic isolation of the protein from BLCa cells which stably knocked down or overexpressed BLACAT3, respectively. WB analysis showed that BLACAT3 knockdown downregulated YBX3 level in the nucleus and upregulated YBX3 level in the cytoplasm (Fig. 7F, I), while BLACAT3 overexpression induced the translocation of YBX3 from the cytoplasm to the nucleus (Fig. 7G, J). Furthermore, we performed IF staining for YBX3 in subcutaneous tumor samples, and found that BLACAT3 knockdown increased YBX3 distribution in the cytoplasm (Fig. 7K). Thus, BLACAT3 may regulate the distribution of YBX3 in cells, and BLACAT3 overexpression induces YBX3 to shuttle into the nucleus.

### BLACAT3 recruits YBX3 to bind the NCF2 promoter, enhance NCF2 transcription, and activates downstream NF-κB signaling pathway

**Fig. 8:**
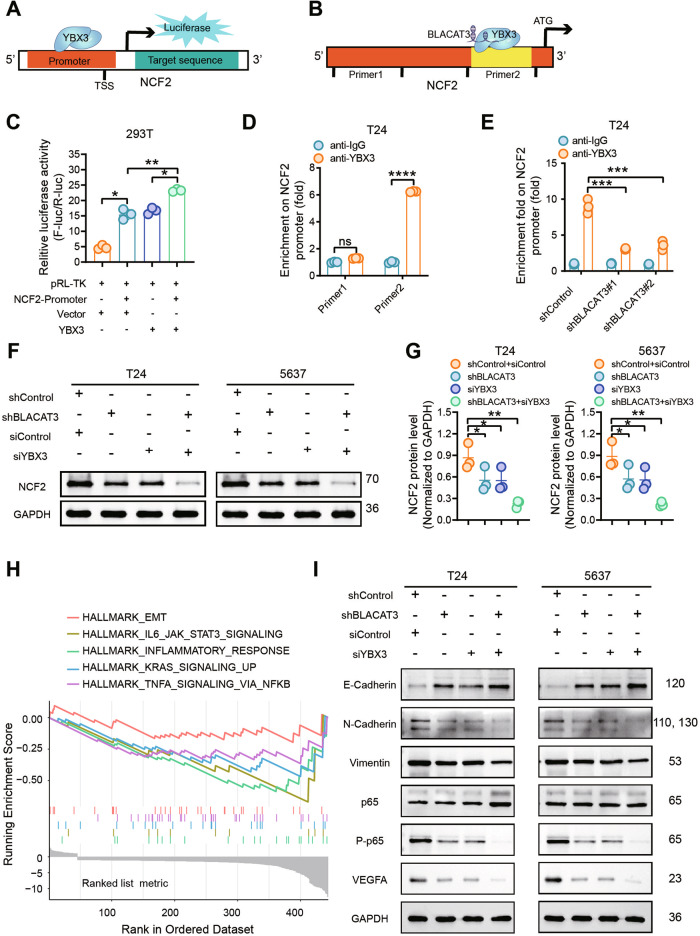
Co-regulation of BLACAT3/YBX3 complex on NCF2 expression by binding NCF2 promoter and enhance NCF2 gene transcription, then activate NF-kB signaling pathway. Dual-luciferase gene reporter assay (**A**, **C**) and ChIP-qPCR assay (**B**, **D**) were performed to verify that BLACAT3 can bind YBX3. **E** ChIP-qPCR was performed to verify BLACAT3/YBX3 complex can bind to NCF2 promoter and regulate the transcription. **F**, **G** WB detected the regulation effects of BLACAT3 and/or YBX3 knockdown on NCF2 protein level. **H** HALLMARK gene set enrichment analysis (GSEA) based on RNA sequencing data revealed typical signaling pathways regulated by BLACAT3. **I** WB verified potential signaling pathways in T24 and 5637 cells. ns no significance. Statistical significance was assessed using two-tailed Student’s *t* test between two groups, *P < 0.05, **P < 0.01, ***P < 0.001, ****P < 0.0001.

It has been reported that YBX3 as a transcription factor (TF) is activated in several cancers [39–41]. To verify whether BLACAT3 regulates the NCF2 expression through YBX3, we reviewed the Chromatin Immunoprecipitation (ChIP) sequencing data of YBX3 in K562 cells via ENCODE integrative analysis [42]. It showed that there were multiple binding peaks for YBX3 and NCF2. The most significant binding peak was located in the NCF2 promoter (sFig. 11A, Supplementary Table 10). Subsequently, we performed dual-luciferase gene reporter (Fig. 8A, C) and ChIP assays (Fig. 8B, D) and verified that YBX3 could bind to NCF2 the promoter. Moreover, BLACAT3 knockdown significantly inhibited the binding affinity between YBX3 and NCF2 promoter (Fig. 8E). To clarify the relationship among BLACAT3, YBX3 and NCF2, we firstly investigated the regulating effect of YBX3 on NCF2. QRT-PCR analysis showed that NCF2 mRNA expression was respectively down- and up-regulated when YBX3 was silenced and overexpressed in T24 (sFig. 11B, C). Next, we silenced YBX3 in T24 and 5637 cells in which BLACAT3 is stably knocked down, and the interfering efficiency of siYBX3 was verified by WB analysis (sFig. 11D–F). WB demonstrated that either BLACAT3 knockdown or YBX3 knockdown could significantly inhibit the protein expression of NCF2, and simultaneous knockdown of BLACAT3 and YBX3 synergistically inhibited NCF2 expression (Fig. 8F, G). Based on our RNA-sequencing data, the Hallmark GSEA results indicated that BLACAT3 knockdown enriched EMT, IL6/JAK/STAT3, inflammatory response, KRAS, and TNFA/NF-κB pro-inflammatory signaling along with other gene sets related to tumor angiogenesis and metastasis (Fig. 8H). WB demonstrated that either knockdown of BLACAT3 or YBX3 significantly upregulated the expression of the epithelial marker E-cadherin, and simultaneous knockdown of BLACAT3 and YBX3 synergistically upregulated E-cadherin level, both in T24 and 5637 cells (Fig. 8I, sFig. 12A, G). For the mesenchymal markers, simultaneous knockdown of BLACAT3 and YBX3 significantly decreased the N-Cadherin expression but expected no effect on Vimentin level (Fig. 8I, *P* > 0.05, sFig. 12B, C, H, I). Notably, WB analyses also showed that when BLACAT3 and YBX3 were simultaneously knocked down, the total protein level of P65 was not changed, while the protein levels of phosphorylated P65 and VEGFA were significantly reduced (Fig. 8I, sFig. 12D–F, J–L), both in T24 and 5637 cells. Furthermore, IHC staining demonstrated that knockdown of BLACAT3 downregulated the expression of phosphorylated P65 and VEGFA in vivo (sFig. 13). Simultaneously, WB assays showed that silencing NCF2 downregulated phosphorylated P65 and VEGFA, while overexpression of BLACAT3 attenuated the downregulated effect (sFig. 14).

## Discussion

M6A RNA methylation, as a reversible epigenetic modification that differentially exists on mRNAs as well as ncRNAs, is involved in tumorigenesis and progression of multiple human malignancies [43]. Previous studies have shown that loss of m6A methylation causes the downregulation of mRNA expression levels [44]. The increased abundance of m6A modifications is one of the reasons for the upregulation of ncRNA expression levels [35, 45]. Therefore, we focused on the 84 lncRNAs with co-upregulated m6A modification abundance and expression level from m6A microarray data. To narrow the filter, we extracted and analyzed TCGA data, which increases the credibility of target prediction. Constructing the prognostic risk model of BLCa patients according to previously reported methodology highlights the clinical translation of BLACAT3 [35, 46, 47].

It is reported that RT4 is the only luminal papillary NMIBC-derived cell line, with all other BLCa cell lines derived from MIBC [48]. Interestingly, no significant difference for both BLACAT3 m6A modification and expression was observed between RT4 and normal urothelial cells, which powerfully verified that the correlation between BLACAT3 m6A modification and expression level likely only exist in MIBC. Our data aligns with previous reports that ALKBH5 plays a leading role in m6A modification in BLCa [49–51].

In vivo, we randomly chose shBLACAT3#2 to investigate the biological function of BLACAT3, based on our observation that shBLACAT3#1 and shBLACAT3#2 exhibit similar inhibitory effects on the malignant biological behavior of BLCa cells in vitro. For constructing tumor models, we chose NSG mouse, which was reported to lack mature T, B, and NK cells and thus exerted a high rate of tumorigenesis [52]. Interestingly, our group failed to construct a tumor model within Balb/c nude mice through the usage of BLCa cells with BLACAT3 knockdown. This is likely due to the silencing of BLACAT3 significantly weakening the colonization ability of BLCa cells. It is clear there is an immense importance of BLACAT3 in the proliferation of BLCa cells.

NCF2/p67phox, the activating subunit of NADPH oxidase, is aberrantly expressed in several genitourinary tumors with clinical significance, including ccRCC [23, 53–55], prostate cancer [56], urothelial carcinoma [20] and testicular cancer [57]. Our data suggests that BLACAT3 positively regulates the expression of NCF2/p67phox. Clinical data showed that NCF2 upregulation is significantly positively correlated with clinicopathological features and poor prognosis of MIBC patients. In addition, cell functional assays revealed that elevated NCF2 promotes BLCa angiogenesis and metastasis.

The subcellular localization of lncRNAs affects their regulatory patterns in tumors [47, 58]. In this study, FISH and qPCR results consistently displayed that BLACAT3 was evenly distributed in the nucleus and cytoplasm. Previously the lncRNAs with subcellular localization-indiscriminate distribution characteristics were also reported [59]. We hypothesize that BLACAT3 can freely pass through nuclear pores and is in dynamic equilibrium on both sides of the nuclear membrane. It is reported that: the interaction between lncRNA and chromosomal DNA occurs in the nucleus, the interaction with miRNA occurs in the cytoplasm, and the interaction with RBPs can occur in both the nucleus and the cytoplasm [38]. Thus, we hypothesized that BLACAT3 regulates downstream genes by interacting with RBPs. Using RNA pull-down and MS we screened potential proteins that bind to BLACAT3. Among the RBP candidates, YBX3 is highly enriched, and it is both RBP and DBP. The dual role of YBX3 can help reveal the regulation mode of BLACAT3 on NCF2.

Y-box binding protein 3 (YBX3) is a member of YBX family, which is also known as cold shock domain protein A (CSDA), DNA binding protein A (DBPA) orzonula occludens 1 (ZO-1)-associated nucleic acid binding protein (ZONAB) [60]. Increasing evidences have shown that YBX3 is aberrantly upregulated in hepatocellular carcinoma [40], pancreatic cancer [61], colon cancer [62] and lung cancer [63]. Using TCGA data analysis we demonstrated no significant difference in the mRNA level of YBX3 between BLCa and normal bladder tissues, and there was no correlation with OS of BLCa patients with YBX3 mRNA expression. However, several studies have confirmed that YBX3 is highly expressed at the protein level and promotes BLCa cell invasion [64, 65]. Unexpectedly, we found that silencing or overexpression of BLACAT3 in BLCa cells had no effect on the protein level of YBX3. Regulation of transcription initiation is performed specifically in the nucleus and is the most direct mode of regulation of gene expression [66]. It is reported that the aberrant nuclear retention of YBX3 facilitates the proliferation of cancer cells [63]. Here, although BLACAT3 has no effect on the overall expression level of YBX3, whether it affects its spatial distribution needs further exploration. Saying this, our WB assay based on nucleocytoplasmic separation and IF staining based on the subcutaneous tumor tissue confirmed that the upregulated BLACAT3 can promote the distribution of YBX3 into the nucleus.

Dual luciferase reporter gene and ChIP assays displayed that YBX3 can bind directly to NCF2 promoter. In addition, BLACAT3 knockdown can decrease the binding of YBX3 to NCF2 promoter. WB analysis further confirmed the cooperative regulation of BLACAT3/YBX3 on NCF2. It has been reported that lncRNA and TF act together on the regulation of target gene promoters [67, 68]. We have shown that BLACAT3 induces YBX3 entry into the nucleus. We speculate that BLACAT3 and YBX3 may combine with the NCF2 promoter in the form of a complex to promote transcription and ultimately upregulate NCF2/p67phox expression. EMT (Epithelial-mesenchymal transition) and NF-κB signaling pathways attracted our attention through GSEA analysis of transcriptomic sequencing data. The role of EMT in various malignant behaviors such as tumorigenesis, metastasis and drug resistance has been widely studied, so we detected the most well-known epithelial marker E-Cadherin and mesenchymal markers N-Cadherin and Vimentin [69]. Our data showed that BLACAT3 can positively promote EMT progression, and that BLACAT3 and YBX3 have a synergistic regulatory effect on EMT, which is consistent with the trend of RNA-seq-based GSEA analysis results, and further verifies the role of BLACAT3 on BLCa metastasis. The role of NF-κB/VEGF signaling pathway in tumor angiogenesis and metastasis has been reported in colon cancer [70], gastric cancer [71], hepatocellular carcinoma [72] and BLCa [73, 74]. Our data showed that knockdown of BLACAT3 or YBX3 significantly inhibited the activation of NF-κB/VEGFA pathway, and simultaneous knockdown of BLACAT3 and YBX3 played a synergistic effect. It is previously reported that ALKBH3 regulates angiogenesis and tumor invasion through NOX2-ROS and Tweak/Fn14/VEGF [19]. We speculate that the upregulated NCF2 in BLCa activates the NF-κB/VEGFA pathway through NADPH oxidase/ROS signaling, and ultimately promote BLCa angiogenesis and metastasis but more data are needed to confirm our hypothesis.

As emerging biological and chemical means have been successfully used to solve the problems of RNA stability, delivery and immunogenicity, RNA therapeutics exhibits broad clinical application scenarios [75]. We are moving towards the goal of developing novel RNA therapeutic targeting BLACAT3 for the treatment of metastatic BLCa. We previously proposed that SLERCC is a promising therapeutics target and that plasma-encapsulated nanoparticles targeting transmembrane metastasis markers may open a new avenue for the treatment in RCC [46]. In addition, the development of new therapeutics targeting RNA m6A modification including enzyme inhibitors has also made progress [76], but there is still a long way to go before clinical application.

Taken together, our data showed that m6A modification contributes to BLACAT3 upregulation by stabilizing RNA structure. We have found that BLACAT3 recruits YBX3 to shuttle into the nucleus, which synergistically enhances NCF2 transcription, and promotes BLCa angiogenesis and hematogenous metastasis by activating downstream NF-κB signaling. Our novel findings will help to develop prognostic prediction tools for BLCa patients and discover new therapeutic biological targets for metastatic BLCa.

## Materials and methods

### Clinical specimens and patient information

Clinical samples and patient information were obtained from two sources for this study. First, publicly available transcriptome sequencing data and corresponding patient clinical data from TCGA. Second, paired tumor and adjacent normal tissues from 107 patients with MIBC who underwent RC in Shanghai Tenth People’s Hospital. Among them, 3 pairs of tumor and adjacent normal tissues were collected from May to September 2020 (Supplementary Table 1). The other 104 pairs of tumor and adjacent normal tissues were collected from January 2012 to December 2017, and information on the demographic and pathological characteristics of patients and clinical samples is provided in Supplementary Table 2. All recruited patients had complete clinicopathological and follow-up data. The study was approved by the Ethics Committee of Shanghai Tenth People’s Hospital (Ethics approval number: SHSY-IEC-4.1/19-210/01). All enrolled patients had signed informed consent forms. All research activities comply with Declaration of Helsinki ethical guidelines.

### Statistics

Under R version 4.0.2 environment, multiple regression analysis and nomogram construction were carried out by rms package, forest chart was drawn by forestplot package, and the ROC curve was produced by pROC package. The other statistical analyses were conducted by SPSS 24.0 and GraphPad Prism 8. The two-tailed Student *t* test was used to compare parameters between two groups of normally distributed data, and the analysis of variance was used to compare multiple groups. The Kaplan-Meier survival analysis method was used to calculate the OS and DSS, and the Log-rank (Mantel-Cox) test was used for statistical analysis. *P < 0.05, **P < 0.01, ***P < 0.001 and ****P < 0.0001 were set as statistically significant, and *P* > 0.05 means no significance.

## Supplementary information

Supplementary information

## Data Availability

The human m6A epitranscriptomic microarray and RNA sequencing data are available in Gene Expression Omnibus: GSE228952, GSE229172. The computer code used in this study is available from the authors if requested by the readers. More details on materials and methods are available in the supplementary information file.
