# Correction: M6A-mediated-upregulation of lncRNA BLACAT3 promotes bladder cancer angiogenesis and hematogenous metastasis through YBX3 nuclear shuttling and enhancing NCF2 transcription

**DOI:** 10.1038/s41388-025-03575-x

**Published:** 2025-09-17

**Authors:** Jinbo Xie, Hui Zhang, Keyi Wang, Jinliang Ni, Xiaoying Ma, Christopher J. Khoury, Viktor Prifti, Brock Hoard, Eric G. Cerenzia, Lei Yin, Houliang Zhang, Ruiliang Wang, Dong Zhuo, Weipu Mao, Bo Peng

**Affiliations:** 1 Department of Urology, Shanghai Tenth People’s Hospital, School of Medicine, Tongji University, Shanghai, 200072 China; 2 Department of Urology, The First Affiliated Hospital of Wannan Medical College (Yijishan Hospital of Wannan Medical College), Wuhu, 241001 China; 3 Department of Laboratory Medicine and Pathobiology, University of Toronto, Toronto, ON M5S 1A1 Canada; 4 Department of Laboratory Medicine, LKSKI-Keenan Research Centre for Biomedical Science, St. Michael’s Hospital, Toronto, ON M5B 1W8 Canada; 5 Department of Anesthesiology and Perioperative Medicine, Shanghai Fourth People’s Hospital, School of Medicine, Tongji University, Shanghai, 200434 China; 6 Department of Urology, Ruijin Hospital, Shanghai Jiaotong University School of Medicine, Shanghai, 200025 China; 7 Department of Urology, Affiliated Zhongda Hospital of Southeast University, Nanjing, 210009 China

**Keywords:** Bladder cancer, Prognostic markers

Correction to: *Oncogene* 10.1038/s41388-023-02814-3, published online 23 August 2023

Following the publication of this article, the authors noted the errors in Figure 3F and 3G. The correct images and fluorescence quantitative statistical graph have now been provided. The authors confirm these corrections do not alter the interpretation of the results or the conclusion of this study.

Former Fig 3:
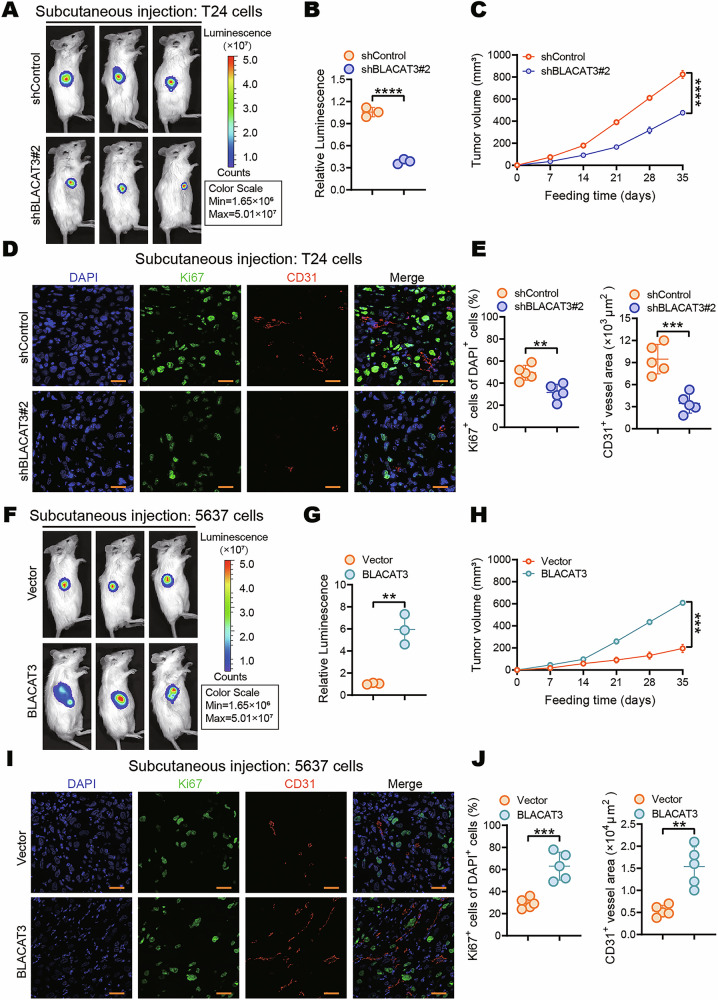


Revised Fig 3:
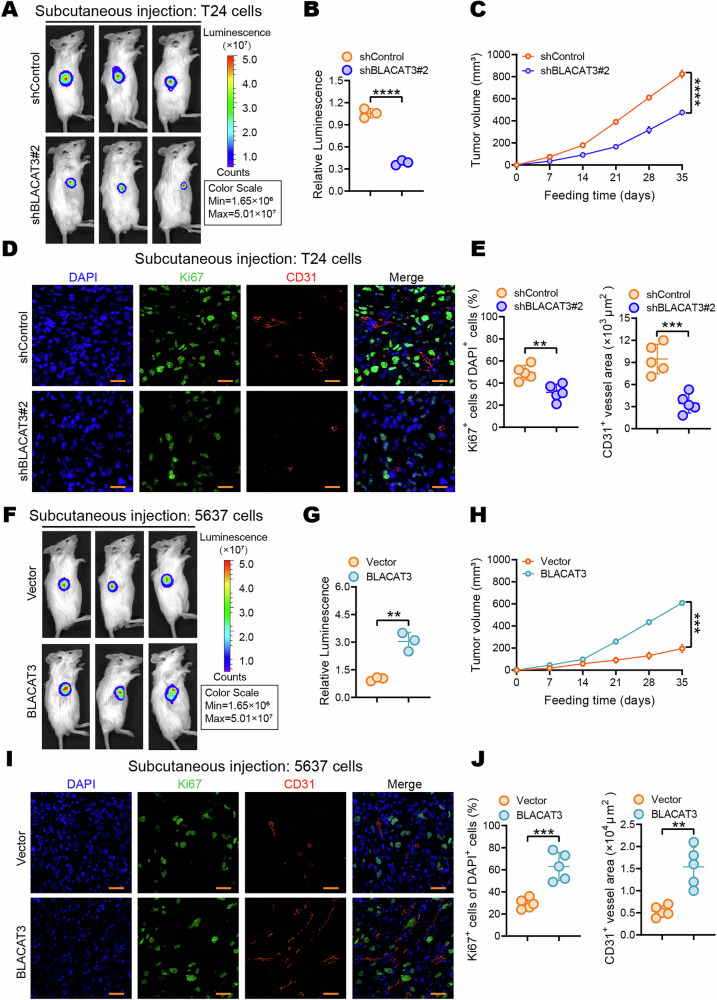


The original article has been corrected.

