# Supplementary material for: M6A-mediated-upregulation of lncRNA BLACAT3 promotes bladder cancer angiogenesis and hematogenous metastasis through YBX3 nuclear shuttling and enhancing NCF2 transcription
Source: Oncogene. 2023 Aug 23;42(40):2956–70. doi: 10.1038/s41388-023-02814-3 (PMC10541332; doi:10.1038/s41388-023-02814-3)
Supplement: Supplementary file 1 — Supplementary information [file 41388_2023_2814_MOESM1_ESM.pdf]

Supplementary Information

This PDF file includes:

Supplementary Materials and Methods

Supplementary Tables 1-11

Supplementary Figure Legends 1-15

Supplementary Figures 1-15

M6A-mediated-upregulation of lncRNA BLACAT3 promotes bladder cancer angiogenesis and hematogenous metastasis through YBX3 nuclear shuttling and enhancing NCF2 transcription

Jinbo Xie[1, 2, 3, 4, 8], Hui Zhang[5, 8], Keyi Wang[1, 8], Jinliang Ni[1, 8], Xiaoying Ma[3, 4], Christopher J. Khoury[3, 4], Viktor Prifti[3, 4], Brock Hoard[3, 4], Eric G. Cerenzia[3, 4], Lei Yin[6], Houliang Zhang[1, 7], Ruiliang Wang[1], Dong Zhuo[2]✉, Weipu Mao[7]✉, Bo Peng[1]✉

SUPPLEMENTARY MATERIALS AND METHODS

Bioinformatics

Bioinformatics analysis were carried out in the R version 4.0.2 environment. In the visualization process, the dplyr package was used for data arrangement and conversion, the RColorBrewer package was used for color setting, scatter plots were drawn by R basic drawing function, volcano maps were drawn by ggplot2 package, Venn diagrams were produced by VennDiagram package, heat maps were drawn by OmicCircos package, bubble charts and bar charts were drawn by ggplot2 package, pie chart was drawn by viridis package, GSEA analysis was carried out by clusterProfiler and enrichplot packages. Adobe Illustrator 2020 was used for post-processing.

Mouse models

A total of 24 male NOD-Prkdcscid Il2rgem1/Smoc mice (1) (Cat. NO.: NM-NSG-001) aged 4 weeks were commercially supplied by Shanghai Model Organisms, Inc (Shanghai, China), and the animal quality identification of SPFcondition was conducted by VRL Laboratories (Suzhou, China) (Certificate NO.: 20170010015248). To ensure statistical significance, the number of mice in each group was set at 3, and 24 mice were randomly assigned to each group. T24 and 5637 cells stably transfected with lentiviruses containing different plasmids were utilized to establish two different mouse models. In subcutaneous xenograft models, 1×10^6 cancer cells suspended in 100 mL of PBS buffer were subcutaneously injected into each mouse. The length and width of the subcutaneous tumor was measured every 7 days, and the tumor volume was calculated by the formula: Volume (mm^3) = 0.5 × L (length) × W^2 (width). On the 28th day after

subcutaneous injection of cells, in vivo imaging of the mice was performed, and the mice were sacrificed on the 35th day. Meantime the subcutaneous tumor tissues were harvested and weighed. The pathology assays were then carried out. In hematogenous metastatic models, each mouse was injected with $3×10^5$ cancer cells in 100 mL of PBS buffer. Live imaging was performed on the 28th day after tail vein injections, and the mice were then sacrificed on the 35th day. The lungs and other organs with suspected metastases were resected, and tissue sections were prepared for H&E, IHC or IF staining. All the animal experiments were approved by Tongji University Laboratory Animal Welfare Ethics Review Working Group Committee (Approval NO.: TJBA01920101).

Cell lines and culture conditions

T24 (RRID: CVCL_0554), 5637 (RRID: CVCL_0126) and UM-UC-3 (RRID: CVCL_1783) cells were cultured in RPMI 1640 medium. J82 (RRID: CVCL_0359) cells were cultured in Minimum Essential Medium (MEM). SV-HUC-1 (RRID: CVCL_3798) cells were cultured in Ham’s F-12K medium. RT4 (RRID: CVCL_0036) cells were cultured in McCoy’s 5a medium. SW780 (RRID: CVCL_1728) and HEK 293T (RRID: CVCL_0063) cells were cultured in Dulbecco’s Modified Eagle’s Medium (DMEM). Human umbilical vein endothelial cells (HUVECs) (RRID: CVCL_2959) were cultured in Endothelial Cell Medium (ECM). The media was pre-treated with 10% fetal bovine serum (FBS; Gibco) and 1% penicillin/streptomycin prior to usage. All the cell lines were purchased from iCell Bioscience Inc. (Shanghai, China) except for SW780, which was purchased from WHELAB (Shanghai, China). Cell line STR authentication and microbial detection was performed by Shanghai Biowing Applied Biotechnology Co. LTD (Shanghai,

China).

Cell transfection

The small interference RNA (siMETTL3, siMETTL14, siWTAP, siFTO, siALKBH5, siYBX3 and siNCF2) oligos were designed and synthesized by IBSBIO (Shanghai, China), and the oligo sequences were listed in Supplementary Table 6. The full-length cDNAs of human ALKBH5 and YBX3 were synthesized and cloned into the pcDNA3.1(+)-3xflag vector. The plasmids which mediated ALKBH5 or YBX3 overexpression were commercially supplied by IBSBIO (Shanghai, China). Transient transfection was performed using Lipofectamine ™ 3000 Transfection Reagent (Catalog # L3000001, Invitrogen, U.S.) in accordance with the manufacturer's protocol.

Lentivirus transduction and screening of stable strains

Lentivirus transduction was performed to establish stable knockdown and overexpression of BLACAT3 BLCa cell strains. Briefly, there were three steps as follows: For lentiviral vector construction, the shRNA sequences that specially target BLACAT3 or the full length of BLACAT3 were cloned into vectors of Plko.1-puro-GFP or CMV-MCS-EF1α-copGFP-T2A-puro (with or without luciferase). The shRNA sequences were listed in Supplementary Table 6. For lentiviral vector package and purification, lentivirus packaging plasmids and vectors of BLACAT3 knockdown or overexpression were con-transfected into the packaging cells (HEK-293T) and incubated for 48 hours, then the supernatants containing virus particles were collected and purified. These two steps were performed by assistant of Zorinbio Technology Co., Ltd (Shanghai, China). For Lentivirus transduction, T24 and 5637 cells were infected by

lentivirus with the presence of polybrene (Catalog # 32160801, Sigma-Aldrich, U.S.), and the stable cell strains were selected by 5 μg/mL of puromycin (Catalog # A1113802, Thermo Fisher Scientific, U.S.) within two weeks.

Cell Counting Kit-8 (CCK-8) assay

CCK-8 reagent was commercially supplied by YEASEN (Catalog#40203ES60, China). T24 or 5637 cells growing in the logarithmic phase were digested to prepare the cell suspension. 2×10^3/100 μL cells per well were added into a 96-well culture plate, then incubated at 37°C. At different time points including 24, 48, 72 and 96 hours, the medium was refreshed, then 10 μL of the CCK-8 solution was added into each well. After incubating for 2 hours at 37°C, the absorbance was detected using microplate reader at wavelength of 450 nm.

5-Ethynyl-2'-deoxyuridine (EdU) assay

Cell-Light™ EdU Apollo567 in vitro Kit was purchased from RiboBio (Catalog # C00054, China). The experiment was performed in accordance with the manufacturer's protocol. Cell nuclei were stained using Hoechst reagent. Pictures were captured by the fluorescent inverted microscope.

Wound-healing assay

BLCa cells were resuspended and cultured in a six-well plate, which should just be confluent overnight. The next day, scratches were made using tips, then cells were washed for 3 times, and refreshed with serum-free medium. Cells were incubated at 37°C. Pictures were captured

by the fluorescent inverted microscope at 0 and 24 hours. Image J software was utilized to measure the percent of wound healed.

Plate Cloning assay

Cells in the logarithmic growth phase were digested and resuspended, then they were seeded in a 6-well plate at a density of 500 cells per well. The cells were cultured at 37°C, and generally the medium was replaced every three days. 14 days later, the culture medium in the six-well plate was removed, and after washing for three times, cells were fixed with formaldehyde solution and stained with 0.1% crystal violet solution. Finally, the colonies were counted and photographed.

HUVEC tube formation assay

200μL Matrigel basement membrane matix (Catalog # 356234, BD Biosciences, U.S.) was added into each well of the 24-well plate and placed in a 37°C incubator for 1h. Next, HUVECs were resuspended in media containing the supernatant from BLCa cells, then 5000 cells per well were seeded in 24-well plates, and cultured at 37℃ for 4 h. Tube formation was photographed under a microscope and counted by Image J software.

Transwell migration assay

Different groups of BLCa cells were pre-suspended in serum-free medium. Each upper chamber was added into 8×10^4 cells, and the lower chamber was added into complete medium containing 10% FBS. After being incubated at 37°C for 12-24 hours, the remaining cells in the

upper chamber were wiped away. Cells penetrating to the outside surface of membrane were fixed with 4% formaldehyde solution, stained with 0.1% crystal violet solution, then photographed under a microscope. Image J software was utilized to count the migrative cells.

RNA stability assays

Actinomycin D (Catalog # A9495, Sigma-Aldrich, U.S.) was used to block the transcription process in the RNA stability assay, with the working concentration 2 μg/mL. After actinomycin D treatment, cell samples were collected at 3 h and 6 h, then RNA was extracted for qRT-PCR analysis.

Subcellular fractionation

RNA and protein subcellular fractionation of BLCa cells were performed using nuclear and cytoplasmic extraction PARIS™ Kit (Catalog # 00738305, Invitrogen, U.S.) in accordance with the manufacturer's constructions. The RNA and protein were respectively prepared for qRT-PCR and WB assays.

RNA extraction and quantitative real-time polymerase chain reaction (qRT-PCR)

Total RNA was extracted from tissues or cells using Trizol reagent (Catalog # T9424, Sigma-Aldrich, U.S.). Reverse transcription was performed using RT reagent Kit (Catalog # RR037Q, Takara, Japan). QRT-PCR analysis was performed using TB Green Premix Ex Taq™ II (Catalog # RR820A, Takara, Japan) in accordance with the manufacturer's constructions. Gene expression was calculated using the $2^{-\Delta\Delta Ct}$ method, with GAPDH or ACTB as the internal

controls. The primer sequences were shown in Supplementary Table 5.

Protein preparation and WB assay

Protein was extracted from tissues or cells using RIPA Lysis Buffer (Catalog # 89900, Thermo Scientific, U.S.). 10% PAGE Gel Fast Preparation Kit was commercially obtained from Epizyme (Catalog # PG112, China) and used to prepare the SDS-PAGE Gels. WB was performed following the previous protocol (2), and the antibodies were listed in Supplementary Table 3.

Dot blot assay

Total RNA was extracted in accordance with the standard procedure. The concentration and OD 260/280 ratio were determined by Nano Drop Spectrophotometer. Total RNA was diluted to 500 ng/μL using DEPC water, and RNA quality was assessed by agarose gel electrophoresis. The RNA was heated to 95°C for 5 min for denaturation of the secondary structure and then immediately cooled on ice for 1 min. 1 μL of denatured RNA was pipetted onto a positively charged nylon membrane and was then placed in a UV cross-linker for cross-linking. Unbound RNA was removed by washing with PBST for 10 min, and blocked with 5% skim milk in PBST for 30 min. The m6A primary antibody (1:1000) was incubated at 4°C overnight, after being washed with PBST four times at 5 min per wash. The secondary antibody was incubated for 1 h at room temperature. After being washed for 5 min four 4 times with PBST again, the nylon membrane was exposed using ECL reagent. The nylon membrane was imaged after being stained with methylene blue for 10 min and washed with ddH2O for 10 min. The antibody information is listed in Supplementary Table 3.

m6A RNA immunoprecipitation (MeRIP)

The EpiQuik CUT&RUN m6A RNA Enrichment (MeRIP) Kit was purchased from Epigentek (P-9018, U.S.). According to the instructions of manufacturer, approximately 1-3 μg of RNA and m6A spike-in control mixture was used in a single experiment. RNA was heated for 5 min at 65℃ and then stored on ice. 300 μL of immunoprecipitation solution system reacted for 2h at 4℃. 20 μL of pre-coated mouse IgG magnetic beads were added into the IP solution system and then incubated at 4℃ overnight on a vertical mixer. The EP tube was placed on the magnetic stand for 10 minutes until the magnetic beads were all adsorbed to the tube wall. The supernatant was collected when the solution became transparent. We then washed with 500 μL of IP buffer three times. For elution, we added 200 μL Elution buffer (100 mM tris, PH 7.4, 1 mM EDTA, 0.05% SDS), 4 μL proteinase, and 2 μL RNase inhibitor for 1h at 50℃. The same volume of phenol-chloroform (phenol: chloroform: isoamyl alcohol = 25: 24: 1) was added into the supernatant and IP samples, shaken vigorously, then rested or 3 min. Following centrifuging for 10 min at 12000g, the supernatant was collected, added into 3 μL glycogen, 20 μL 3 M NaAC and 50 μL iced absolute ethanol, then was placed at -20℃ for 30 min. After being centrifuged at 15000 g for 10 min, the system was washed using 80% ethanol, then the sample was dissolved in 20 μL ddH20.

m6A single-base validation using ligase-based method

Single-base validation using the ligation-amplification method was performed as described previously (3). Briefly, 1000 ng of processed RNA was first prepared. The mixture A (20 nM

probe L, 20 nM probe R, 1× ligation buffer (NEB) and 1000 ng treated RNA) was configured. After configuration, the mixture was incubated for 3 minutes at 85°C, then cooled to 35°C for 10 minutes. Mixture B was added after configuration to a volume of 20 μL. Then it was left in room temperature for 10 min and set aside. QRT-PCR was performed on the sites of Ct, respectively. All data were calculated by the formula: m6A% = [(2 - Ct unmethylated) - (2 - Ct methylated site)] / (2 - Ct unmethylated) x 100 %. The probes and PCR primers used in this experiment were respectively listed in Supplementary Table 4 and Supplementary Table 5.

Fluorescence in situ hybridization (FISH)

The RNA FISH probe mix was purchased from RiboBio (Guangzhou, China). All FISH probe sequences are unavailable due to commercial patent protection. Multiple probes were designed to target BLACAT3, 18S rRNA and U6 sequences to improve the fluorescence signal-to-noise ratio. To provide optimal signal intensity, each set of 15-20 probes could cover the entire length of the RNA molecule, and each probe carried multiple fluorophores. FISH probes were stored in RNase-free stock solution at -20°C, protected from light. The experimental steps were implemented in accordance with the protocol provided by the manufacturer. Cell nuclei were stained with DAPI and images were collected using the confocal microscope ZEISS LSM 900 (Carl Zeiss AG, Germany).

RNA pull-down assay and MS

The pre-experimental preparations included the extraction of total protein, the preparation of BLACAT3 cDNA template, and biotin labeling. First, a combined reaction was performed

between streptavidin magnetic beads and labeled sense or anti-sense probes. Then, the RNA-magnetic beads were labeled and protein was bound. Master mix of RNA-protein binding reaction was prepared and added into the above protein complex and incubated at room temperature for 2 hours. Finally, the RNA-binding protein complexes were rinsed and eluted for silver staining SDS-PAGE and MS analysis.

RNA immunoprecipitation (RIP) assay

RNA-binding protein immunoprecipitation was performed after obtaining cell lysates. 300 μL supernatant was pipetted into an EP tube. 2 μg anti-ZONAB antibody was added and the sample was incubated at 4°C for 2 hours. Next, we added 50 μL of protein A/G magnetic bead suspensions and incubated overnight at 4°C. Then, we place the EP tube on the magnetic stand, and discarded the supernatant. We added 500 μL RIP Wash Buffer, put the EP tube on the magnetic rack after spin washing, discarded the supernatant, and washed four times. RNA purification was then carried out. Here, we added 1 μL proteinase K and 150 μL Wash Buffer to the magnetic bead-antibody complex and incubated at 55℃ for 30 min to remove the protein. After incubation, we placed the EP tube on a magnetic stand, aspirated the supernatant into a new EP tube, and added 250 μL of RIP Wash Buffer. Then we used phenol: chloroform: isoamyl alcohol to extract RNA. Finally, we removed the supernatant, dried the RNA precipitate and then added 20 μL DEPC water to dissolve the RNA. After concentration quantification using Nanodrop, the RNAs were reverse transcribed into cDNA and performed qRT-PCR analyzing. The antibody and primers used in this experiment were respectively listed in Supplementary Table 3 and Supplementary Table 5.

Dual-luciferase reporter assay

The reporter gene plasmid was constructed and supplied by IBSBIO (Shanghai, China). The detection was performed according to the instructions of the reporter gene detection kit. Cell lysate was obtained after transfection of HEK 293T cells. The fully lysed lysate was centrifuged at 10,000-15,000 rpm for 3-5 min. After centrifugation, the supernatant was transferred to a new EP tube for subsequent detection. The fluorescence detection was conducted according to the instrument manual. We added 20 μL of sample and 20 μL of Firefly Luciferase Assay Reagent into the tube and measured the RLU after thorough mixing. Then, we added 20 μL of the prepared Renilla Luciferase Assay working solution to the sample solution and fully mixed. Finally, the RLU was measured. The activation degree of the reporter gene is determined by the ratio obtained between the sample RLU and standard RLU value. The promoter sequences of NCF2 were listed in Supplementary Table 10.

Chromatin Immunoprecipitation (ChIP) assay

The binding of ZONAB in the promoter region of NCF2 gene was analyzed by ChIP experiments in T24 cells under different treatment conditions. ChIP experiments were performed using Anti-ZONAB antibody, and two pairs of qRT-PCR primers were designed according to the binding site (one pair for the binding site region and one pair for the distal control region) to analyze ZONAB on the NCF2 gene promoter. First, in vitro DNA-protein conjugate cross-linking was carried out. For nucleus preparation, 5 mL of TNT Buffer (10 mM Tris-HCl, 10 mM NaCl, 0.2% Trition X-100, 1× protease inhibitor, pH 8.0) were added, and incubated on ice for 10 min. Then

the buffer was centrifuged at 2000 rpm, 4°C, for 5 min, discarding the supernatant, and cell nucleus pellet was stored at -80°C for later use. Nuclear lysis and ultrasonic shearing of chromatin were performed, and the OD value was detected after ultrasonication. Next, immunoprecipitation was performed. After washing, the immunoprecipitation was eluted and cross-linked. The supernatant was discarded, and 30μL ddH2O was added after drying. The relative enrichment level of ZONAB in the promoter region of NCF2 gene was detected by qRT-PCR. The antibody and primers used in this experiment were respectively listed in Supplementary Table 3 and Supplementary Table 5.

Haematoxylin and eosin (H&E) staining

The tissues were sectioned for subsequent staining after the steps of sampling, fixing, dehydration and wax-embedding. H&E staining was performed as previously described. All the slides were scanned using pannoramic digital pathology scanners (3D HISTECH, Hungary), and pictures were processed using CaseViewer (3D HISTECH, Hungary).

Immunofluorescence (IF) and Immunohistochemical (IHC) staining

The experiments were performed based on the protocols previously described. Before the experiment started, the paraffin slides were sequentially processed by dewaxing, antigen retrieval, and serum blocking. For IF staining, it generally went through the steps of primary antibody reaction, secondary antibody reaction, reaction amplification, addition of phalloidin, DAPI counterstaining of nuclei, and sealing. For IHC staining, an additional diaminobenzidine reaction is added on the basis of the IF staining. All the slides were scanned using pannoramic

digital pathology scanners (3D HISTECH, Hungary), and pictures were processed using CaseViewer (3D HISTECH, Hungary). All the antibodies used in IF and IHC stainings were listed in Supplementary Table 3.

REFERENCES

1. Li F, Zhang H, Wang W, Yang P, Huang Y, Zhang J, et al. T cell receptor β-chain-targeting chimeric antigen receptor T cells against T cell malignancies. Nature communications. 2022;13(1):4334.

2. Chang M, Gao F, Chen J, Gnawali G, Wang W. MDM2-BCL-X(L) PROTACs enable degradation of BCL-X(L) and stabilization of p53. Acta materia medica. 2022;1(3):333-42.

3. Zhang Z, Chen LQ, Zhao YL, Yang CG, Roundtree IA, Zhang Z, et al. Single-base mapping of m(6)A by an antibody-independent method. Science advances. 2019;5(7):eaax0250.

SUPPLEMENTARY TABLES

Supplementary Table 1. Patient characteristics for m6A microarray analysis (n = 3).

Patient ID	Gender	Age(y)	Histopathological Type	Grade	TNM stage
P1(D****9)	Male	71	Urothelium carcinoma	III	T2bN0M0
P2(D****6)	Male	67	Urothelium carcinoma	III	T4aN0M0
P3(D****4)	Male	63	Urothelium carcinoma	III	T3aN2M0

Supplementary Table 2. Characteristics of patient from local hospital (n = 104).

Variable	Frequency	BLACAT3 expression		Pearson Chi-square	*P* value
		Low	High		
Total	104	52	52		
Gender					
Male	73	39	34	1.149	0.284
Female	31	13	18		
Age(y)					
46-75	68	40	28	6.118	0.013
76-90	36	12	24		
Marital status					
Married	63	35	28	1.973	0.160
Solo living	41	17	24		
Tumor grade					
Low (I/II)	4	1	3	0.260*	0.610
High (III/IV)	100	51	49		
Tumor stage					
Tis/T1	2	2	0	0.510*	0.475
T2-T4	102	50	52		
L.N. status					
Negative	98	52	46	4.422*	0.035
Positive	6	0	6		
TNM stage					
Ois/I/II	86	49	37	9.674	0.002
III/IV	18	3	15		

*Correction for continuity

Supplementary Table 3. Antibody list

Antibody Name	Manufacturer	Catalog Number	Application
anti-m6A	Synaptic Systems	202003	m6A IP
anti-m6A	ZEN-BIOSCIENCE	517924	m6A dot blot (1:1000)
anti-ALKBH5	Abcam	ab195377	WB (1:1000)
anti-GAPDH	Abcam	ab181602	WB (1:10000)
anti-Ki67	Proteintech	27309-1-AP	IF (1:16000)
anti-CD31	Proteintech	28083-1-AP	IF (1:2000)
anti-NCF2	Abcam	ab109366	IHC (1:100), WB (1:1000)
anti-YBX3/ZONAB	Bethyl Laboratories	A303-070A	WB (1:1000), IP (1:100)
anti-Lamin B	Abcam	ab32535	WB (1:500)
anti-E-Cadherin	Proteintech	20874-1-AP	WB (1:5000)
anti-N-Cadherin	Abcam	ab245117	WB (1:1000)
anti-Vimentin	Abcam	ab92547	WB (1:1000)
anti-NF-kB p65	Abcam	ab16502	WB (1:1000), IHC (1:100)
anti-Phospho-NF-κB p65	Cell Signaling Technology	#3033	WB (1:1000), IHC (1:800)
anti-VEGFA	Abcam	ab46154	WB (1:1000)
anti-VEGFA	Abcam	Ab1316	IHC (1:100)

Supplementary Table 4. Probes used in this study

Probe name	Sequences	Application
NC	L1: po4GCGGAACCACTATACACAGCCGAAGAACCTTTACC<br>R1: TCTGGTGGCATCACTCATCTGAACATCCCAAGGrTrU	Single-base mapping of m6A
BLACAT3	L1: po4CCGGGCGCCCATCAATAACAACACGGTAGCAGGAGT<br>R1: ACCTGGACCACTTGGAACATATTGCTACATCAGCCCCArGrU	

Supplementary Table 5. Primer sequences used in this project.

Gene name	Primer sequences	Application
ENST00000439898.1	F: 5'- GCCTGGTCAGTGGCATAG-3'<br>R: 5'- GATTGGGGTCAGTTTTGG-3'	MeRIP qRT-PCR
BLACAT3	F: 5' -CCCAACAATCTGTGTTTAACAA-3'<br>R: 5'- ATCCCGAGATCATCCAAATC-3'	MeRIP qRT-PCR
BLACAT3	F: 5' -CGACCTCTGACACATAGGATCTG-3'<br>R: 5' -ACAGAGGAATTGGAAAAGAAGCTC-3'	qRT-PCR
METTL3	F: 5' -TTGTCTCCAACCTTCCGTAGT-3'<br>R: 5' -CCAGATCAGAGAGGTGGTGTAG-3'	qRT-PCR
METTL14	F: 5' -AGTGCCGACAGCATTGGTG-3'<br>R: 5' -GGAGCAGAGGTATCATAGGAAGC-3'	qRT-PCR
WTAP	F: 5' -CTTCCCAAGAAGGTTCGATTGA-3'<br>R: 5' -TCAGACTCTCTTAGGCCAGTTAC-3'	qRT-PCR
ALKBH5	F: 5' -CGGCGAAGGCTACACTTACG-3'<br>R: 5' -CCACCAGCTTTTGGATCACCA-3'	qRT-PCR
FTO	F: 5' -ACTTGGCTCCCTTATCTGACC-3'<br>R: 5' -TGTGCAGTGTGAGAAAGGCTT-3'	qRT-PCR
GAPDH	F: 5' -GGACCTGACCTGCCGTCTAG-3'<br>R: 5' -GTAGCCCAGGATGCCCTTGA-3'	qRT-PCR
U6	F: 5'-CGCTTCGGCAGCACATATACTA-3'<br>R: 5'-TCACGAATTTGCGTGTCATCCT-3'	qRT-PCR
YBX3	F:5'-ACCGGCGTCCCTACAATTAC-3'<br>R:5'-GGTTCTCAGTTGGTGCTTCAC-3'	qRT-PCR
NCF2	F: 5'-CCAGAAGCATTAACCGAGACAA-3'<br>R: 5'-CCTCGAAGCTGAATCAAGGC-3'	qRT-PCR
NCF2 Primer1	F: 5'-GGCATAGCGGGAGAAGAGTAA-3'<br>R: 5'-TATCCCACCCTGACACTCAGA-3'	ChIP qRT-PCR
NCF2 Primer2	F: 5'-AGTGAGCAAGGAATTCATTCAAG-3'<br>R: 5'-CCTTGATTTCCACACCCTTGT-3'	ChIP qRT-PCR

Supplementary Table 6. Sequences of siRNAs and shRNAs oligos used in this project

siRNAs/shRNAs	Sense (Forward oligos)	Antisense (Reverse oligos)
siControl	5'-UUCUCCGAACGUGUCACGUTT-3'	5'-ACGUGACACGUUCGGAGAATT-3'
siMETTL3#1	5'-GCAAGUAUGUUCACUAUGAAA-3'	5'-UCAUAGUGAACAUACUUGCAG-3'
siMETTL3#2	5'-GACUGCUCUUUCCUUAAUAUU-3'	5'-UAUUAAGGAAAGAGCAGUCUU-3'
siMETTL14#1	5'-GGAUGAGUUAAUAGCUAAAUC-3'	5'-UUUAGCUAUUAACUCAUCCUU-3'
siMETTL14#2	5'-GCAUUGGUGCCGUGUUAAAUU-3'	5'-UUUAACACGGCACCAAUGCUU-3'
siWTAP#1	5'-GCUUUGGAGGGCAAGUACAUU-3'	5'-UGUACUUGCCCUCCAAAGCUU-3'
siWTAP#2	5'-CUAAGAGAGUCUGAAGAAAUU–3'	5'-UUUCUUCAGACUCUCUUAGUU–3'
siFTO#1	5'-GGUUCACAACCUCGGUUUAGU-3'	5'-UAAACCGAGGUUGUGAACCGG-3'
SiFTO#2	5'-GGAUGACUCUCAUCUCGAAGG-3'	5'-UUCGAGAUGAGAGUCAUCCUC-3'
siALKBH5#1	5'-GCUGCAAGUUCCAGUUCAAGC-3'	5'-UUGAACUGGAACUUGCAGCCG-3'
siALKBH5#2	5'-GCGCCGUCAUCAACGACUACC-3'	5'-UAGUCGUUGAUGACGGCGCUG-3'
siYBX3#1	5'-GCAAUAAAGUGGAAGACUAAC-3'	5'-UAGUCUUCCACUUUAUUGCUU-3'
siYBX3#2	5'-GCAGAGCAGUGCUGAGUAAUU-3'	5'-UUACUCAGCACUGCUCUGCUG-3'
siYBX3#3	5'-GGAUGGAGUUCCUGUGGAAGG-3'	5'-UUCCACAGGAACUCCAUCCGG-3'
siNCF2#1	5'-CACUCAAGGUGCACUACAAGU-3'	5'-UUGUAGUGCACCUUGAGUGUG-3'
siNCF2#1	5'-CGGUAGUCAUGAAGACUCAGC-3'	5'-UGAGUCUUCAUGACUACCGUG-3'
siNCF2-3	5'-CCAAGGAGUAAGUACAAAUUU-3'	5'-AUUUGUACUUACUCCUUGGUU-3'
shScramble	5'-CCTAAGGTTAAGTCGCCCTCG-3'	5'-CGAGGGCGACTTAACCTTAGG-3'
shBLACAT3#1	5'-CCGGCATAGGATCTGGAGATTAAAGCTCGAGCTTTAATCTCCAGATCCTATGTTTTTG-3'	5'-AATTCAAAAAGGGTATAGTGGTTCCGCAACCCTCGAGGGTTGCGGAACCACTATACCC-3'
shBLACAT3#2	5'-CCGGGGGTATAGTGGTTCCGCAACCCTCGAGGGTTGCGGAACCACTATACCCTTTTTG-3'	5'-AATTCAAAAAGGGTATAGTGGTTCCGCAACCCTCGAG GGTTGCGGAACCACTATACCC-3'
shBLACAT3#3	5'-CCGGGCTGCTCTGCTAATAATTAGGCTCGAGCCTAATTATTAGCAGAGCAGCTTTTTG-3'	5'-AATTCAAAAAGCTGCTCTGCTAATAATTAGGCTCGAG CCTAATTATTAGCAGAGCAGC-3'

Supplementary Table 7. Experiment Workflow of Human m6A-mRNA&lncRNA Epitranscriptomic Microarray (Supplied by Aksomics Inc, China)

RNA Sample QC

The purity and amount of total RNA samples were determined with NanoDrop ND-1000. Results were provided in Sample QC report.

↓

m6A Immunoprecipitation

Total RNAs were immunoprecipitated with anti-N6-methyadenosine(m6A) antibody. The immunoprecipitated "IP" fraction contained enriched m6A methylated RNAs, and the supernatant " Sup" fraction contained unmodified RNAs.

↓

Two-color RNA Labeling

"IP" and "Sup" RNAs were amplified as cRNAs and labeled with Cy5 and Cy3 respectively using Arraystar Super RNA Labeling Kit.

↓

Array Hybridization

Cy3 and Cy5 labeled cRNAs were combined together and hybridized to Arraystar Human mRNA&lncRNA Epitranscriptomic Arrays (8x60K, Arraystar) at 65°C for 17 hours in an Agilent Hybridization Oven.

↓

Array Scanning

After washing, slides were scanned with Agilent Scanner G2505C.

↓

Data Processing and Analysis

Supplementary Table 8. Flowchart of m6A Microarray Data Analysis (Supplied by Aksomics Inc, China)

Raw Data Extraction

Data was extracted using Agilent Feature Extraction software. The probe signals passing "P" or "M" QC flags in at least 1 sample were retained for further analysis.

"m6A methylation level" and "RNA expression level" Calculation

IP (Cy5-labelled) and Sup (Cy3-labelled) intensities were normalized by the RNA spike-in calibration controls. The m6A methylation levels were calculated for the percentage of modified RNA of a transcript. The RNA expression levels were calculated by total of modified and not modified RNA of a transcript.

"m6A quantity" Calculation

IP (Cy5-labelled) intensities were normalized as relative intensities by the RNA spike-in calibration controls. The normalized IP intensities represent the "m6A quantity" of the transcript comparable across samples.

Differentially m6A-methylated or expressed mRNAs, lncRNAs and other ncRNAs

Differentially m6A-methylated or expressed mRNAs, lncRNAs and other ncRNAs based on "m6A methylation level", "m6A quantity" and "RNA expression level" passing fold change and statistical significance cutoffs were identified and compiled.

Further data analysis

Hierarchical clustering heatmap analysis was performed for differentially m6A-methylated or expressed RNAs. Differentially m6A-methylated or expressed mRNAs were analyzed for GO and pathway enrichment.

Supplementary Table 9. Full-length cDNA sequences of two candidate lncRNAs from m6A Microarray data

> BLACAT3/ENST00000591116.5/LINC01535-204 cDNA: lncRNA 815bp

AGGCGCACGGCGGGUAUAGUGGUUCCGCAACCUUGGGAUGUUCGGCUGAGCGCCU
GCCUAACAUGGGAGCGCCUGCCUAACCCUCCUGACUGGAGGGUCAGCGGCGGGGA
GUCCUGGCCUUCAAUCGGCCAUCGCCACGGCAGAAACUGGGGCCCGGGCUGCGUC
CAGGCCCUUCCCCGUCCGUCCACAGGUCUCAUGGGCGCCCGGACUGGGGCUGAUG
UAGUUUCCCGACCUCUGACACAUAGGAUCUGGAGAUUAAAGCUGCUCUGCUAAUAA
UUAGGUUCCCACCUCUCAAGAUCAUUAGGAGCUUCUUUUCCAAUUCCUCUGUUUUG
CAUGCAUUGGAAGGAUCUGGAGCUCAGGACCCACUACCUCCCCGGCCCACUGCAA
GCUUGAACAUUUGAUGGUGGACACCCCUCCCUGACUUUGUAUUAUGAUUCACAGGC
UGCUCCCCAGAAUACUGAAGAAGAAACUCUAGGAGUGAGACCCAACAAUCUGUGUU
UAACAAGUCCCCAGGCCCCUGGAUCCUCCUGGAAUCCCUGAUCCCUGUUGCUCCC
UGGCGGAGUUUUCUAGUCCCUCUUACUUGAAGCAUGUCUUUCAGAGCCCGGCACC
ACCCAAGUUGGGUAUCUGAUACCCCAUCAGCCUGAGUCUCUUAAAGGCCUUUAUAC
ACAUAUGUCUACACAUAGGGAUUUGGAUGAUCUCGGGAUCCCACAUCCUCGCUGUC
CCCUGUCCCCCCACAACAUCCCACACCAAUACCUUUCUGAAGUUUUCUAGUCCCUC
CUUUUUGUUUGUGGUCCUUAAAGCCCAGCCCCAUGCCU

> ENST00000439898.1 cDNA: lncRNA 2625bp

GGGGTCGGCGCCGCGGAGGCGGGGAGGGAGGGAGGGTTGGAGGGTTGAGCCAGA
GACCCGGGACCCCGTCCCCGCACACCTGCCAGTGCCCCGTAGCTTCGGCCGCCGGC
ACTGGCAGGAGATGAAAGGCTGCTGCCGCCCGGTCGGAAGGACATCGGCGCCCCCC
AGGCCCGGTCCCCGCCCCAGTTCCTCGGGCCTTTCCTGCTGCCCCTGCCTGCGAGG
GCCGACGACACGGAGAACAGGATCCTGCGCCCAACCCAGGTCCCCGCCTTCTTTCAG
AGGCCCAGGCCTGGACCCCGCTGAGCCGCAGATGTGCGAGCAGGAGCGCCAGAGC
CCCGATGCCCGCCCAGCAGGAAGCGGGCGGGAGATGGTTCCTTCCTTCTGTCCTGAG
GGGGAACCCTGCACAGAGGGACCATTGAGGGCCTGGCATTGTCTGCCTAACTCACCC
AGTGCCTCCCTCCCTGGGTGGGCCATGCGGGGCCTTGACAGGATTGCCCTGGTGCC
GTCTTGGCAGTGGGTCTGGGTGGGATCCTGGGGGCAGGGCTTCCCTGAGTGCAGAC
AGCTAGGCCTCCACCTGCCCCGGCCTCCCACCCAGGCTCAGATTTCCAGGGCATAAG
GCTCCATTGTCCCAGCACTGGTGGAGGCGGCCTGTCAATTCAGCCTTGTGTTTGGTG
GTTGGGAAATTCCCAGCCATGGGGGGCTGCAGGCAGGAAGGGGCTGCCCAGGTGTC
CTGCACCCCAACTGAAGGGACTCCATGAGGTTGGTTCCTGGGCATCCCCTGCTGCCT
GGAGCTGTCCCAGGCTGGACCTCAACCATTCATCAACCCTCAGGAGCAGTTGGGTGA
GGAGCACCAGAAATTCAATGCTCCCTGGCGCTGCATCCCCAGAGCCCTCCCAGCCTA
AGAAGCCCCATCTTTCTGTCTCCACGCATGGAGAACTGCAGCTGTGAGGCCCAGGAC
CCTTAGCAGGACATGCAGAGCTGGGCAGGGACCCAGGCTCATGCTCCCAGCGTGGG
GTGAGTTGTCTCCAGCCTGTGGAGACTGCCATGAAGTTGATCTGCCTCCCAGAGGGC
CTGGCCCACTTGAAATAATTGCTCCGGCTACTGATGTGGTGGGAACTTTGGTATTTTTA
ACCCATTTGGGGGGTGGGGGAGCAGCTAGGAAGAGAGAGGCAAGCTTTCAGAGTCA
GAGAGGCCTGAGAGAGGAGAGTAGAGGGAAACTCAGTGAGGAGGAGCCAGGCAGGC
TGCCTCGGTAGTTCCCCAGGCCTAGACACCCCCCCTGTACCACCCCCTGTCCCAGCA
GGTAGGTGCAGACCTAGATGCCAGGTGCAGAAGGGGGAAAGGGCCCTCTCCAGGGT
TACAGCAGGGATCACCGAGGCTGCAGGGGCTGCCAAGGCCTGGAAGAAGTCCCATGT

TCCAGGGAGCCCCATGGCTTCTGATGTCAGGAAAACTTAGTCCTCTCAGTTCCCCAGA
ATCATTTCACCCCACCCCACCCAAACTGAGTGGCAAACCAGTTGAGTAGAGAATACAA
GCCCTGACTCCAGCTGCCTGGTCAGTGGCATAGCCAGCCAAGTCCTAGCAACCCTAG
GAGTCAGGGAGTCAGGGAGGAGGCAAGGACAAGACTACAGTATTGTTTGGCTGAGTT
CTGGGTCTGGCCCCACTCCCCAAAACTGACCCCAATCTCTGTGTCTGCTGCCCTAAAA
AGAGACCCTGGGGCTGGGTGTGGTGGCTCACGCCTGTAATCCTAGCACTTTGGGAGG
CCAAGGTGGGCGGATCACTTGAGATCAGGAGTTCAAGACCAGCCTGGCCAACATGGT
GAAACCCCGTCTCTACTAAAATACAAAAATTAGCTGGGCATGATGACGGGTGCCTGTAA
TCCCAGCTACTCAGGAGGCTGAAACAGGAGAATCACTTGAACCCAGGAGACGGTGGT
TGCAGTGAGCCAAGATTGTGCCACTGCACTCTAGCCTAGGTGGCTGAGCGAGACTCC
ATCTCAAAAAAATAAATAAAAGGAGACCCTGACTGGATGTAGTGGCTCATGCCTTAATCC
CAGCACTTTTGGAGGCCAAGGCAGGAGGATCACTTGAGGCCAAAAGTTTGAGACCAG
CCTGGGCAACATAGCAAGACCCCGTCTCTTAAAAACAAAAGATCCTAGCGGTCCTCATC
TCTACCATGGACTACCAGAGGGAAGGCAGCACCTCTCATCACCCAGGGGGATGGCCT
CCAGTCAGCTGGGTGGGGAACACAGAGCCCTGCCCAGAGGCTTGAACCTGGCACCA
CAGGGGTCTGGAATTACACAGAAGACGGGTGACAGCCAAGGTGGATCATGAACGGTG
AGAAGTCCAGCAGGTGACAAGGGGAAGGGTCTAAAGGGTGGAGGGCACAGCGCAAG
CAAAGTCTTGGCAACAAAAGAGCTAATGCATCCCAGAAATGGGGCAGGTGGAGTACTG
GAAGCTACACCAAGCTTCAGAGTGGTCCTGTGGCCTCGGTGTGGTAGCTCAGGCCTAT
AATTCCAACACTTTGGGAGGCTGAGGCAGGAGGAGGATAACTTGAACCCAGGAGTTCA
AGATCAGCCTGGGCAACATAGTGAGACCTCCATTTTTACAAAAAATACAAAAATTAACTG
TG

Supplementary Table 10. The promoter sequences of NCF2

ggcatagcgggagaagagtaagcagcagagtttgtatgggattgtgaaggaaagttctggggataaggctagaaaagtcttg
gcagtcagattaaggagagttctgagtgtcagggtgggataataggagggcaaaaacattttttaaaaatgaagtctaatttagc
tacaaatccatttggttttctcaggaataattaatgtgctattcaagtcctgattatttattcactagatggaatagcaatacctctttctg
ccatcactgctacctgcaggtcatttaccaatgatttctaatttcttttagaatgtatcccttcatcatgtattgagtatctgtgctagaga
tcctagaatgaaaaggcatggtcccaacttccaaggagcccatgaattgagcagataaagagataaattgcagtaagtatgat
gcaagtttttgggtaaaggtggtacaaaggagaggaaaattctcctactacccagagttcagggctagatttagcacagttggtg
acatttaaactgggctttgaaagatgagtaggagtttgccaggcaaagaagagagctgggaaggccttgtaaacacaggaa
aacaatctggacaaagacacacatgtcttcttaattctcacaccttcaatcctacaagcactgtggagtatacatttttaagttttattt
tcaggcttttaggggaagcttggtgataatgagtgaattctttcagttgctacaaaacaagagtttgcatctaagtatttgaccccca
taaacatgcattttacctaaaaagaagcctggattttagcaagccaaattggctatacccttttacccccagttattcctctgggagg
ttttactgatttttacttttctggaaaagcaaggaaagaactcttaaatctgtttttttcagagactagtaaatccattagtcatcagtgca
ggactttcatctcttcaatttttcactggaagatgggagaagtcataattcactgtcatgtggctcccaattttgacatttgcaggaaa
ttggtcccctctgaacttcatgaatgttcataaaagttatttcggctgggcacggtggctcactcctgtaatcccagcactttgaggg
gaggccgaggaggccgaatcacctgaggtcaggagttcgagaccatcagcctggccaacatggtgaaaccccatctctact
aaaaatacaaaaattagcagggcgtggtggcaggcttctgtaatcccagctactcaggaggctgaggcaggagaattgcttg
aacccaggaggcagaggttgcaatgagctgagatcgtgtcactgcattccagcctgggcaacaagagtgaaactccgtctca
aaaaaaaaaaaaaaaaagttatttcaggcccagatcagatggatctccaaagattatcctgtctcaaaaatttgttaatgataat
ttcagctcaagataaacactaacatattaatacagcctatgggattacaaaaaagtgagcaaggaattcattcaagactggaa
agaattatcctctcatctgacactcccatttaacaagggtgtggaaatcaaggcccagtgacacaggattttttcTCCATCAct
ttgtaagccaggaacctctggctggtgacaccctgcccaggcctcagtgggccacactacctgctgcaggagatggcctgccc
actcggcctgggtggggagttttattgagtgatgaaaacagctttcagtggagaggggatgcaggggtggttcccctacccaaa
ggcaggaaagtccccccagtgcgggtgagtctggggcttttatgggctcagaataggggaggggcaggacataggtagtact
ggaaaaggcaacattcaattggttaaaaggcattagtcagaaataattaattgggaaagagcagacaaacaggaacagaa
gttctcactctaggtcacgggtttcatttgggaccagtagcCTAGTCTTTCAGCCTTCAGGCTGTTTTTGGCT
TGAAGCTCTCTTGGCCTCCTAGTTTCTACCTAATCATGTCCCTGGTGGAGGCCATCAGC
CTCTGGAATGAAGGGGTGCTGGCAGCGGACAAGAAGGACTGGAAGGGAGCCCTGGA
TGCCTTCAGTGCCGTCCAGGACCCCCACTCCCGGATTTGCTTCAACATTGGCT

Supplementary Table 11. BLACAT3-linked proteins in RNA pull-down assay

	Accession	Description
1	Q5VTE0	Putative elongation factor 1-alpha-like 3
2	P26599	Polypyrimidine tract-binding protein 1
3	P27824	Calnexin
4	P14866	Heterogeneous nuclear ribonucleoprotein L
5	Q9Y6M1	Insulin-like growth factor 2 mRNA-binding protein 2
6	P04843	Dolichyl-diphosphooligosaccharide-protein glycosyltransferase subunit 1
7	Q9P0L0	Vesicle-associated membrane protein-associated protein A
8	P16989	Y-box-binding protein 3
9	Q12906	Interleukin enhancer-binding factor 3
10	Q00839	Heterogeneous nuclear ribonucleoprotein U
11	P04844	Dolichyl-diphosphooligosaccharide-protein glycosyltransferase subunit 2
12	Q7Z2W4	Zinc finger CCCH-type antiviral protein 1
13	Q12905	Interleukin enhancer-binding factor 2
14	P31327	Carbamoyl-phosphate synthase [ammonia] mitochondrial
15	Q9NZB2	Constitutive coactivator of PPAR-gamma-like protein 1
16	Q96AG4	Leucine-rich repeat-containing protein 59
17	P22626	Heterogeneous nuclear ribonucleoproteins A2/B1
18	P41091	Eukaryotic translation initiation factor 2 subunit 3
19	Q2VIR3	Eukaryotic translation initiation factor 2 subunit 3B
20	P43243	Matrin-3
21	P13010	X-ray repair cross-complementing protein 5
22	Q15785	Mitochondrial import receptor subunit TOM34
23	O95292	Vesicle-associated membrane protein-associated protein B/C
24	P09914	Interferon-induced protein with tetratricopeptide repeats 1
25	Q08211	ATP-dependent RNA helicase A
26	Q07065	Cytoskeleton-associated protein 4
27	O95786	Probable ATP-dependent RNA helicase DDX58
28	Q13765	Nascent polypeptide-associated complex subunit alpha
29	E9PAV3	Nascent polypeptide-associated complex subunit alpha muscle-specific form
30	P60468	Protein transport protein Sec61 subunit beta
31	P30443	HLA class I histocompatibility antigen A-1 alpha chain
32	P30455	HLA class I histocompatibility antigen A-36 alpha chain
33	P46977	Dolichyl-diphosphooligosaccharide-protein glycosyltransferase subunit STT3A
34	P14868	Aspartate--tRNA ligase cytoplasmic
35	Q99729	Heterogeneous nuclear ribonucleoprotein A/B
36	P02545	Prelamin-A/C
37	P62280	40S ribosomal protein S11
38	P62244	40S ribosomal protein S15a
39	Q53GQ0	Very-long-chain 3-oxoacyl-CoA reductase
40	O15173	Membrane-associated progesterone receptor component 2
41	P05062	Fructose-bisphosphate aldolase B
42	P04083	Annexin A1

43	P62913	60S ribosomal protein L11
44	P51114	Fragile X mental retardation syndrome-related protein 1
45	O95793	Double-stranded RNA-binding protein Staufen homolog 1
46	P39656	Dolichyl-diphosphooligosaccharide-protein glycosyltransferase 48 kDa subunit
47	P07814	Bifunctional glutamate/proline--tRNA ligase
48	Q15233	Non-POU domain-containing octamer-binding protein
49	Q9GZZ8	Extracellular glycoprotein lacritin
50	P23284	Peptidyl-prolyl cis-trans isomerase B
51	Q6PIU2	Neutral cholesterol ester hydrolase 1
52	Q9NQC3	Reticulon-4
53	P16615	Sarcoplasmic/endoplasmic reticulum calcium ATPase 2
54	P10620	Microsomal glutathione S-transferase 1
55	Q15392	Delta (24)-sterol reductase
56	P14625	Endoplasmin
57	O95758	Polypyrimidine tract-binding protein 3
58	O00442	RNA 3'-terminal phosphate cyclase
59	O00425	Insulin-like growth factor 2 mRNA-binding protein 3
60	P29558	RNA-binding motif single-stranded-interacting protein 1
61	P31689	DnaJ homolog subfamily A member 1
62	P61009	Signal peptidase complex subunit 3
63	P07205	Phosphoglycerate kinase 2
64	Q9NPA0	ER membrane protein complex subunit 7
65	Q9UN86	Ras GTPase-activating protein-binding protein 2
66	Q96DA0	Zymogen granule protein 16 homolog B
67	Q13283	Ras GTPase-activating protein-binding protein 1
68	P23246	Splicing factor proline- and glutamine-rich
69	P51991	Heterogeneous nuclear ribonucleoprotein A3
70	Q12797	Aspartyl/asparaginyl beta-hydroxylase
71	P00387	NADH-cytochrome b5 reductase 3
72	P49755	Transmembrane emp24 domain-containing protein 10
73	P49368	T-complex protein 1 subunit gamma
74	O96005	Cleft lip and palate transmembrane protein 1
75	Q13325	Interferon-induced protein with tetratricopeptide repeats 5
76	Q15046	Lysine--tRNA ligase
77	O75396	Vesicle-trafficking protein SEC22b
78	Q8TCJ2	Dolichyl-diphosphooligosaccharide-protein glycosyltransferase subunit STT3B
79	P62906	60S ribosomal protein L10a
80	P33121	Long-chain-fatty-acid--CoA ligase 1
81	P07237	Protein disulfide-isomerase
82	P29692	Elongation factor 1-delta
83	A0FGR8	Extended synaptotagmin-2
84	P0CF74	Immunoglobulin lambda constant 6
85	P0DOY2	Immunoglobulin lambda constant 2
86	P0CG04	Immunoglobulin lambda constant 1

87	P0DOY3	Immunoglobulin lambda constant 3
88	B9A064	Immunoglobulin lambda-like polypeptide 5
89	Q14258	E3 ubiquitin/ISG15 ligase TRIM25
90	P17987	T-complex protein 1 subunit alpha
91	Q9Y3I0	tRNA-splicing ligase RtcB homolog
92	Q16850	Lanosterol 14-alpha demethylase
93	P15531	Nucleoside diphosphate kinase A
94	Q07666	KH domain-containing RNA-binding signal transduction-associated protein 1
95	Q96AE4	Far upstream element-binding protein 1
96	Q96QA5	Gasdermin-A
97	Q9NZI8	Insulin-like growth factor 2 mRNA-binding protein 1
98	Q00266	S-adenosylmethionine synthase isoform type-1
99	P12956	X-ray repair cross-complementing protein 6
100	Q9BTV4	Transmembrane protein 43
101	P62266	40S ribosomal protein S23
102	P63244	Receptor of activated protein C kinase 1
103	P68400	Casein kinase II subunit alpha
104	Q8NEV1	Casein kinase II subunit alpha 3
105	P01591	Immunoglobulin J chain
106	Q5JQF8	Polyadenylate-binding protein 1-like 2
107	Q4VXU2	Polyadenylate-binding protein 1-like
108	Q9H361	Polyadenylate-binding protein 3
109	P11940	Polyadenylate-binding protein 1
110	P12268	Inosine-5'-monophosphate dehydrogenase 2
111	Q9BSJ8	Extended synaptotagmin-1
112	Q15365	Poly(rC)-binding protein 1
113	Q15366	Poly(rC)-binding protein 2
114	P57721	Poly(rC)-binding protein 3
115	Q9NZZ3	Charged multivesicular body protein 5
116	Q12904	Aminoacyl tRNA synthase complex-interacting multifunctional protein 1
117	Q70UQ0	Inhibitor of nuclear factor kappa-B kinase-interacting protein
118	P49257	Protein ERGIC-53
119	Q15125	3-beta-hydroxysteroid-Delta (8) Delta (7)-isomerase
120	P51648	Aldehyde dehydrogenase family 3 member A2
121	Q9UI42	Carboxypeptidase A4
122	Q9P035	Very-long-chain (3R)-3-hydroxyacyl-CoA dehydratase 3
123	Q01130	Serine/arginine-rich splicing factor 2
124	Q9BRL6	Serine/arginine-rich splicing factor 8
125	P60842	Eukaryotic initiation factor 4A-I
126	P50990	T-complex protein 1 subunit theta
127	Q6NZI2	Caveolae-associated protein 1
128	Q13200	26S proteasome non-ATPase regulatory subunit 2
129	Q9Y3E5	Peptidyl-tRNA hydrolase 2 mitochondrial
130	Q99878	Histone H2A type 1-J

131	Q16777	Histone H2A type 2-C
132	Q9BTM1	Histone H2A.J
133	Q93077	Histone H2A type 1-C
134	P0C0S8	Histone H2A type 1
135	Q7L7L0	Histone H2A type 3
136	P16435	NADPH--cytochrome P450 reductase
137	P11586	C-1-tetrahydrofolate synthase cytoplasmic
138	P78371	T-complex protein 1 subunit beta
139	P25788	Proteasome subunit alpha type-3
140	P07910	Heterogeneous nuclear ribonucleoproteins C1/C2
141	O15523	ATP-dependent RNA helicase DDX3Y
142	O00571	ATP-dependent RNA helicase DDX3X
143	P46779	60S ribosomal protein L28
144	Q13586	Stromal interaction molecule 1
145	P04732	Metallothionein-1E
146	P02795	Metallothionein-2
147	P80297	Metallothionein-1X
148	Q8N339	Metallothionein-1M
149	P13640	Metallothionein-1G
150	Q8TCT9	Minor histocompatibility antigen H13
151	P12236	ADP/ATP translocase 3
152	P05141	ADP/ATP translocase 2
153	P12235	ADP/ATP translocase 1
154	Q15717	ELAV-like protein 1
155	Q5T750	Skin-specific protein 32
156	Q16563	Synaptophysin-like protein 1
157	Q15155	Nodal modulator 1
158	P69849	Nodal modulator 3
159	Q5JPE7	Nodal modulator 2
160	P09913	Interferon-induced protein with tetratricopeptide repeats 2
161	P61254	60S ribosomal protein L26
162	Q9UNX3	60S ribosomal protein L26-like 1
163	P01037	Cystatin-SN
164	P01036	Cystatin-S
165	Q9Y4X1	UDP-glucuronosyltransferase 2A1
166	Q6UWM9	UDP-glucuronosyltransferase 2A3
167	P06133	UDP-glucuronosyltransferase 2B4
168	O75795	UDP-glucuronosyltransferase 2B17
169	Q96L21	60S ribosomal protein L10-like
170	P27635	60S ribosomal protein L10
171	O15347	High mobility group protein B3
172	P41252	Isoleucine--tRNA ligase cytoplasmic
173	Q86UP2	Kinectin
174	P62899	60S ribosomal protein L31

175	P51659	Peroxisomal multifunctional enzyme type 2
176	P22735	Protein-glutamine gamma-glutamyltransferase K
177	P61769	Beta-2-microglobulin
178	P35610	Sterol O-acyltransferase 1
179	Q9H299	SH3 domain-binding glutamic acid-rich-like protein 3
180	P26038	Moesin
181	P38159	RNA-binding motif protein X chromosome
182	O14879	Interferon-induced protein with tetratricopeptide repeats 3
183	P50402	Emerin
184	P05166	Propionyl-CoA carboxylase beta chain mitochondrial
185	O76024	Wolframin
186	P06748	Nucleophosmin
187	P63000	Ras-related C3 botulinum toxin substrate 1
188	P60763	Ras-related C3 botulinum toxin substrate 3
189	P15153	Ras-related C3 botulinum toxin substrate 2
190	P67812	Signal peptidase complex catalytic subunit SEC11A
191	P01833	Polymeric immunoglobulin receptor
192	Q00610	Clathrin heavy chain 1
193	P00326	Alcohol dehydrogenase 1C
194	P00966	Argininosuccinate synthase
195	P60903	Protein S100-A10
196	P10809	60 kDa heat shock protein mitochondrial
197	P61803	Dolichyl-diphosphooligosaccharide-protein glycosyltransferase subunit DAD1
198	P40926	Malate dehydrogenase mitochondrial
199	Q96L46	Calpain small subunit 2
200	P04632	Calpain small subunit 1
201	Q96EP5	DAZ-associated protein 1
202	P62854	40S ribosomal protein S26
203	Q5JNZ5	Putative 40S ribosomal protein S26-like 1
204	Q16378	Proline-rich protein 4
205	P50991	T-complex protein 1 subunit delta
206	P62851	40S ribosomal protein S25
207	Q16658	Fascin
208	O43402	ER membrane protein complex subunit 8
209	Q7KZF4	Staphylococcal nuclease domain-containing protein 1
210	Q92820	Gamma-glutamyl hydrolase
211	Q9NR12	PDZ and LIM domain protein 7
212	Q92499	ATP-dependent RNA helicase DDX1
213	O95819	Mitogen-activated protein kinase kinase kinase kinase 4
214	P30050	60S ribosomal protein L12
215	O75844	CAAX prenyl protease 1 homolog
216	Q13404	Ubiquitin-conjugating enzyme E2 variant 1
217	P84090	Enhancer of rudimentary homolog
218	Q8N5K1	CDGSH iron-sulfur domain-containing protein 2

219	P15311	Ezrin
220	P63162	Small nuclear ribonucleoprotein-associated protein N
221	P14678	Small nuclear ribonucleoprotein-associated proteins B and B'
222	Q9UKS7	Zinc finger protein Helios
223	O75635	Serpin B7
224	P08240	Signal recognition particle receptor subunit alpha
225	Q99442	Translocation protein SEC62
226	Q14739	Delta (14)-sterol reductase
227	Q8N684	Cleavage and polyadenylation specificity factor subunit 7
228	Q9P1U1	Actin-related protein 3B
229	P61158	Actin-related protein 3
230	P07099	Epoxide hydrolase 1
231	P05090	Apolipoprotein D
232	O15260	Surfeit locus protein 4
233	P53999	Activated RNA polymerase II transcriptional coactivator p15
234	Q8IZH2	5'-3' exoribonuclease 1
235	Q04637	Eukaryotic translation initiation factor 4 gamma 1
236	Q9HB71	Calcyclin-binding protein
237	Q01813	ATP-dependent 6-phosphofructokinase platelet type
238	P17858	ATP-dependent 6-phosphofructokinase liver type
239	P08237	ATP-dependent 6-phosphofructokinase muscle type
240	Q96JB3	Hypermethylated in cancer 2 protein
241	A8MT65	Zinc finger protein 891
242	Q9NZD8	Maspardin
243	P09210	Glutathione S-transferase A2
244	Q16772	Glutathione S-transferase A3
245	P08263	Glutathione S-transferase A1
246	Q7RTV2	Glutathione S-transferase A5
247	P47914	60S ribosomal protein L29
248	Q8NFW8	N-acylneuraminate cytidylyltransferase
249	P43307	Translocon-associated protein subunit alpha
250	P47895	Aldehyde dehydrogenase family 1 member A3
251	P00352	Retinal dehydrogenase 1
252	P30837	Aldehyde dehydrogenase X mitochondrial
253	P05091	Aldehyde dehydrogenase mitochondrial
254	O94788	Retinal dehydrogenase 2
255	Q9H9S3	Protein transport protein Sec61 subunit alpha isoform 2
256	P61619	Protein transport protein Sec61 subunit alpha isoform 1
257	Q6VVX0	Vitamin D 25-hydroxylase
258	P61353	60S ribosomal protein L27
259	P46781	40S ribosomal protein S9
260	P06396	Gelsolin
261	Q9Y3A5	Ribosome maturation protein SBDS
262	Q6P4A8	Phospholipase B-like 1

263	Q00341	Vigilin
264	Q9BVI4	Nucleolar complex protein 4 homolog
265	Q13148	TAR DNA-binding protein 43
266	O75132	Zinc finger BED domain-containing protein 4

SUPPLEMENTARY FIGURE LEGENDS

Supplementary Figure 1. Biological characteristics and clinical significance of BLACAT3. A and B. Volcano plots showing lncRNAs with differential m6A modification levels (**A**) and expression levels (**B**) in 3 pairs of MIBC tissues and adjacent normal tissues. Abs(log_2FC) > 0.585 and $P < 0.05$ were set as the thresholds. **C.** Diagram showed the chromosomal distribution of BLACAT3 in human BLCa tumor tissues and adjacent normal tissues. **D-G.** GO and KEGG analyses by ranking pearson correlation coefficient between BLACAT3 and all mRNAs in m6A epitranscriptomic microarray matrix. Representative GSEA results related to cell devision, protein binding, HIF-1 signaling and metabolism were shown. **H and I.** Evaluation of the protein-coding potential of BLACAT3 by Coding Potential Calculator 2 (CPC2, http://cpc2.gao-lab.org/). ACTB and GAPDH are recognized protein-coding genes. HOTAIR and BLACAT2 are widely confirmed lncRNAs. **J.** QRT-PCR was conducted to detect BLACAT3 expression in BLCa tumor tissues and adjacent normal tissues (n = 104). **K.** Kaplan-Meier survival curves showed the effect of BLACAT3 expression on the DSS of BLCa patients (Log-rank (Mantel-Cox) test, $P < 0.05$). Total of 104 BLCa patients were divided into high expression group and low expression group by the median value of relative BLACAT3 expression. **L.** Receiver operating characteristic curves demonstrated the prognostic accuracy of nomograms at 1, 2, 3, and 4 years. **M.** Calibration curves for OS of 1-year, 2-year, 3-year and 4-year verified the satisfied fitting degree of this model. Statistical significance was assessed using two-tailed Student's t test between two groups, $^{****}P < 0.0001$. Abbreviations: MIBC, Muscle-invasive bladder cancer; GO, Gene Ontology; KEGG, Kyoto Encyclopedia of Genes and Genomes; GSEA, Gene Set Enrichment Analysis.

Supplementary Figure 2. A. QRT-PCR assay was performed to determine BLACAT3 expression in different BLCa cell lines (RT4, UM-UC-3, 5637, T24, J82 and SW780) and normal urothelial cell line (SV-HUC-1). **B.** Anti-m6A dot blot assay was performed to examine the overall m6A modification level of three BLCa cell lines (5637, UM-UC-3 and T24) and normal urothelial cell line (SV-HUC-1). **C.** QRT-PCR assay was used to verify the knockdown efficiency of three main RNA methyltransferases (METTL3, METTL14 and WTAP) and two typical RNA demethylases (ALKBH5 and FTO) in 5637 cells. **D.** MeRIP and qRT-PCR assay were

performed to detect BLACAT3 m6A modification proportion after knockdown of five m6A regulatory enzymes in 5637 cells. **E.** WB assay was conducted to investigate the protein level of ALKBH5 in different cell lines. Statistical significance was assessed using two-tailed Student's t test between two groups, *P < 0.05, **P < 0.01, ***P < 0.001, ****P < 0.0001. Abbreviations: ns, no significance; qRT-PCR, Quantitative Real-Time Polymerase Chain Reaction; MeRIP, Methylated RNA Immunoprecipitation; WB, Western blots.

Supplementary Figure 3. The effects of ALKBH5 on BLCa angiogenesis and migration in vitro. A-B. CCK-8 assay was conducted to determine the regulatory effect of ALKBH5 on the proliferation of 5637 and T24 cells. **C-H.** HUVEC tube formation assay and transwell migration assay were performed to detect the effects of ALKBH5 knockdown (**C-E**) and overexpression (**F-H**) on the pro-angiogenic and migration of BLCa cells. Scale bars: 2 mm (black lines), 500 µm (orange lines). Statistical significance was assessed using two-tailed Student's t test between two groups, *P < 0.05, ****P < 0.0001. Abbreviations: Cell Counting Kit-8.

Supplementary Figure 4. Effects of BLACAT3 knockdown on proliferation of BLCa cells. A-B. QRT-PCR assays were conducted to verify the knockdown efficiency of BLACAT3 in T24 and 5637 cells. **C and D.** NSG mice were sacrificed on research endpoint (day 35) after subcutaneous injection of stably BLACAT3 knockdown T24 cells, and the subcutaneous tumors were photographed and weighed. **E.** The subcutaneous tumor was fixed and embedded and then stained with H&E. Scale bars: 1.2mm (black lines), 30 µm (orange lines). **F-H.** EdU assay was used to determine the effect of BLACAT3 knockdown on the proliferation of T24 and 5637 cells. Scale bars: 30 µm. **I-K.** Plate cloning assay was performed to detect the effect of BLACAT3 knockdown on sphere formation in T24 and 5637 cells. Statistical significance was assessed using two-tailed Student's t test between two groups, *P < 0.05, ****P < 0.0001. Abbreviations: qRT-PCR, Quantitative Real-Time Polymerase Chain Reaction; NSG, NOD-SCID IL-2receptor Gamma null; H&E, Haematoxylin and Eosin; EdU, 5-Ethynyl-2'-deoxyuridine.

Supplementary Figure 5. Effects of BLACAT3 overexpression on proliferation of BLCa cells. A-B. QRT-PCR assay was used to verify the overexpression efficiency of BLACAT3 in

T24 and 5637 cells. **C-D.** NSG mice were sacrificed on the research endpoint (day 35) after subcutaneous injection of 5637 cells stably BLACAT3 overexpression, and the subcutaneous tumors were photographed and weighed. **E.** The subcutaneous tumor was fixed，embedded and then stained with H&E. Scale bars: 1.2mm (black lines), 30 μm (orange lines). **F-H.** EdU assay was conducted to detect the effect of BLACAT3 overexpression on the proliferation of T24 and 5637 cells. Scale bars: 30 μm. **I-K.** Plate cloning assay was performed to examine the effect of BLACAT3 overexpression on sphere formation in T24 and 5637 cells. Statistical significance was assessed using two-tailed Student's t test between two groups, $*P < 0.05$, $**P < 0.01$, $****P < 0.0001$. Abbreviations: qRT-PCR, Quantitative Real-Time Polymerase Chain Reaction; NSG, NOD-SCID IL-2receptor Gamma null; H&E, Haematoxylin and Eosin; EdU, 5-Ethynyl-2'-deoxyuridine.

Supplementary Figure 6. Effects of BLACAT3 knockdown on the migration potential of BLCa cells. A-D. Wound healing assay was used to detect the effect of BLACA3 knockdown on the migration of T24 and 5637 cells. Scale bars: 5 mm. Statistical significance was assessed using two-tailed Student's t test between two groups, $****P < 0.0001$.

Supplementary Figure 7. Effects of BLACAT3 overexpression on angiogenesis and migration of BLCa cells in vitro. A-H. HUVEC tube formation assay, transwell migration assay and wound healing assay were performed to examine the effect of BLACAT3 overexpression on the angiogenesis and migration of T24 (**A-D**) and 5637 (**E-H**) cells. Scale bars: 2 mm (black lines), 500 μm (red lines), 5 mm (orange lines). Statistical significance was assessed using two-tailed Student's t test between two groups, $***P < 0.001$, $****P < 0.0001$. Abbreviations: HUVEC, Human Umbilical Vein Endothelial Cell.

Supplementary Figure 8. Effects of BLACAT3 overexpression on BLCa cell hematogenous metastasis in vivo. A and B. In vivo imaging was performed on day 28 after tail vein injection of 5637 cells with stable BLACAT3 overexpression into NSG mice, and fluorescence quantitative statistics were performed on representative images. **C and D.** NSG mice were sacrificed on the 28th day after tail vein injection model was constructed, and gross

samples of kidney and lung were isolated, fixed, embedded and sliced for H&E staining. Scale bars: 1.2 mm (black lines), 30 μm (orange lines). Statistical significance was assessed using two-tailed Student's t test between two groups, *****P* < 0.0001. Abbreviations: NSG, NOD-SCID IL-2receptor Gamma null; H&E, Haematoxylin and Eosin.

Supplementary Figure 9. RNA-sequencing analysis. A. Volcano plot showed the proportion of differentially expressed genes after BLACAT3 knockdown. **B.** GO analysis of biological functions enriched by differentially expressed genes after BLACAT3 knockdown. **C.** QRT-PCR assay was performed to detect NCF2 expression in clinical BLCa tumor tissues and adjacent normal tissues (n = 104). **D.** WikiPathways analysis of signaling pathways enriched based on differentially expressed genes after BLACAT3 knockdown. Statistical significance was assessed using two-tailed Student's t test between two groups, *****P* < 0.0001. Abbreviations: GO, Gene Ontology; qRT-PCR, Quantitative Real-Time Polymerase Chain Reaction.

Supplementary Figure 10. YBX3 identification by MS and functional enrichment analysis. A and B. GO analysis of biological functions enriched by BLACAT3-binding proteins identified by MS. **C.** KEGG and WikiPathways analysis of signaling pathways enriched by BLACAT3-binding proteins. **D.** The top 15 RNA Binding proteins pulled down by BLACAT3. **E.** Differential expression profile of mRNAs between 411 BLCa tumor tissues and 19 normal bladder tissues in the TCGA-BLCa dataset showed that YBX3 was not differentially expressed. **F.** The relationship between the mRNA level of YBX3 and BLCa patient OS was analyzed based on the TCGA data (*P* > 0.05). Abbreviations: ns, no significance; GO, Gene Ontology; KEGG, Kyoto Encyclopedia of Genes and Genomes; TCGA, The Cancer Genome Atlas; OS, Overall Survival.

Supplementary Figure 11. YBX3 binds to NCF2 promoter and regulate transcription. A. ChIP-sequencing data (https://www.encodeproject.org/) in K562 cells showed the sequence site of YBX3 binding to NCF2. **B.** QRT-PCR assay was performed to verify the knockdown efficiency of YBX3, and detected the effect of YBX3 knockdown on NCF2 mRNA expression in T24 cells. **C.** QRT-PCR assays were performed to verify the overexpression efficiency of YBX3,

and detect the effect of YBX3 overexpression on NCF2 mRNA expression in T24 cells. **D-F.** WB verified the knockdown efficiency of YBX3 in T24 and 5637 cell lines. Statistical significance was assessed using two-tailed Student's t test between two groups, **P* < 0.05, ***P* < 0.01, *****P* < 0.0001. Abbreviations: ChIP, Chromatin immunoprecipitation; qRT-PCR, Quantitative Real-Time Polymerase Chain Reaction.

Supplementary Figure 12. Statistical histogram of WB bands. Statistical significance was assessed using two-tailed Student's t test between two groups. **P* < 0.05, ***P* < 0.01, ****P* < 0.001. Abbreviation: ns, no significance.

Supplementary Figure 13. BLACAT3 promotes BLCa proliferation and metastasis via acivating NF-κB signaling pathway. A. IHC asaay was conducted to investigate the effect of BLACAT3 on NF-κB signaling pathway in subcutaneous xenograft model. **B.** IHC asaay was conducted to investigate the effect of BLACAT3 on NF-κB signaling pathway in hematogenous metastasis model. Abbreviation: IHC, immunohistochemistry.

Supplementary Figure 14. BLACAT3/NCF2 axis can activate NF-κB signaling pathway. A. WB assay was conducted to investigate the effect of BLACAT3/NCF2 axis on NF-κB signaling pathway. **B.** Statistical histogram of WB bands. Statistical significance was assessed using two-tailed Student's t test between two groups. **P* < 0.05, ***P* < 0.01, ****P* < 0.001. Abbreviation: ns, no significance.

Supplementary Figure 15. Mechanism diagram by which BLACAT3 promotes BLCa tumor angiogenesis and metastasis. M6A modification contributes to BLACAT3 upregulation by stabilizing RNA structure. BLACAT3 induces YBX3 to shuttle into the nucleus, synergistically enhances NCF2 transcription, and promotes BLCa angiogenesis and metastasis via activating NF-κB signaling pathway.

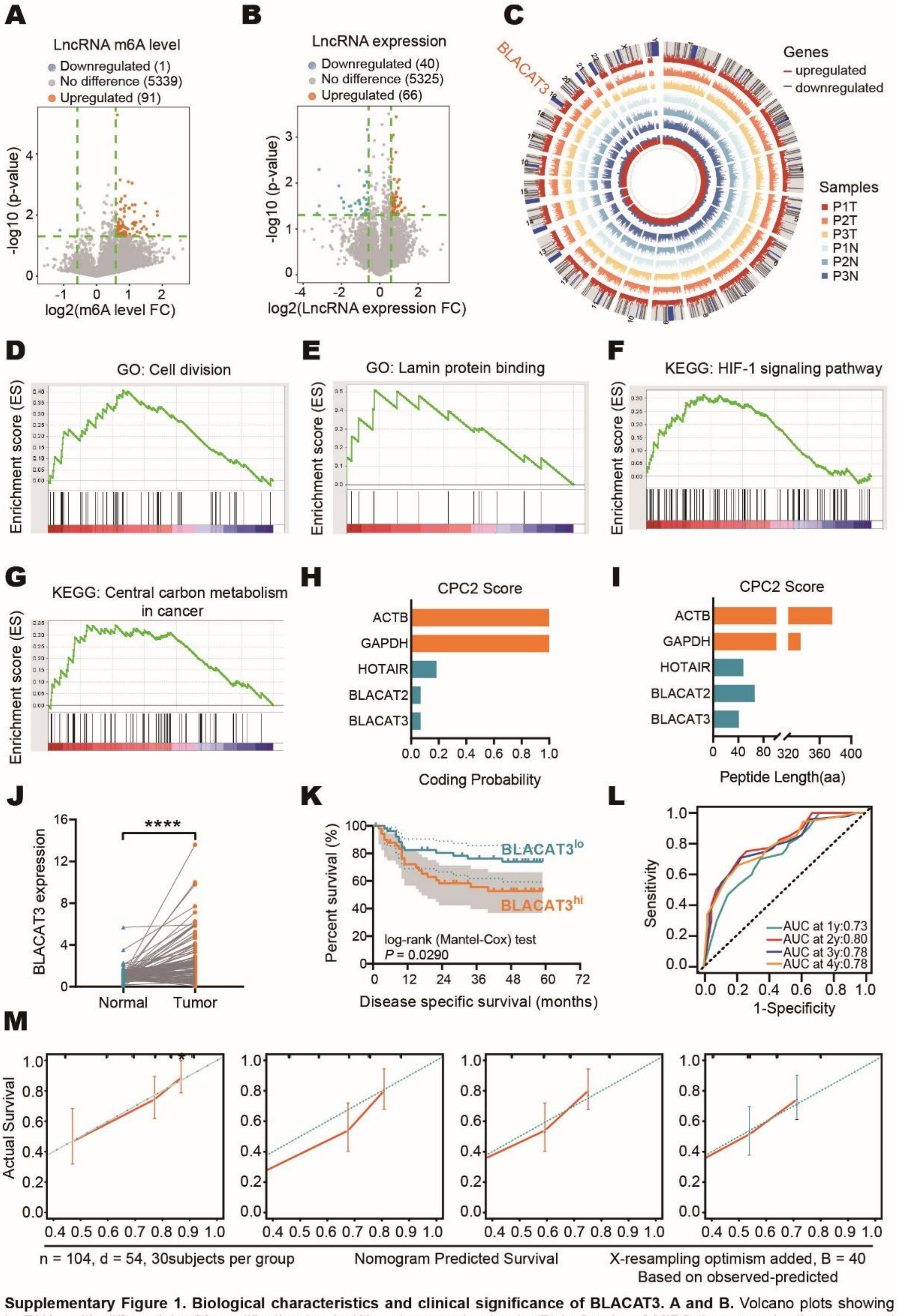

Supplementary Figure 1. Biological characteristics and clinical significance of BLACAT3. A and B. Volcano plots showing lncRNAs with differential m6A modification levels (A) and expression levels (B) in 3 pairs of MIBC tissues and adjacent normal tissues. Abs(log2FC) > 0.585 and $P < 0.05$ were set as the thresholds. **C.** Diagram showed the chromosomal distribution of BLACAT3 in human BLCa tumor tissues and adjacent normal tissues. **D-G.** GO and KEGG analyses by ranking pearson correlation coefficient between BLACAT3 and all mRNAs in m6A epitranscriptomic microarray matrix. Representative GSEA results related to cell devision, protein binding, HIF-1 signaling and metabolism were shown. **H and I.** Evaluation of the protein-coding potential of BLACAT3 by Coding Potential Calculator 2 (CPC2, http://cpc2.gao-lab.org/). ACTB and GAPDH are recognized protein-coding genes. HOTAIR and BLACAT2 are widely confirmed lncRNAs. **J.** QRT-PCR was conducted to detect BLACAT3 expression in BLCa tumor tissues and adjacent normal tissues (n = 104). **K.** Kaplan-Meier survival curves showed the effect of BLACAT3 expression on the DSS of BLCa patients (Log-rank (Mantel-Cox) test, $P < 0.05$). Total of 104 BLCa patients were divided into high expression group and low expression group by the median value of relative BLACAT3 expression. **L.** Receiver operating characteristic curves demonstrated the prognostic accuracy of nomograms at 1, 2, 3, and 4 years. **M.** Calibration curves for OS of 1-year, 2-year, 3-year and 4-year verified the satisfied fitting degree of this model. Statistical significance was assessed using two-tailed Student's t test between two groups, ****$P < 0.0001$. Abbreviations: MIBC, Muscle-invasive bladder cancer; GO, Gene Ontology; KEGG, Kyoto Encyclopedia of Genes and Genomes; GSEA, Gene Set Enrichment Analysis.

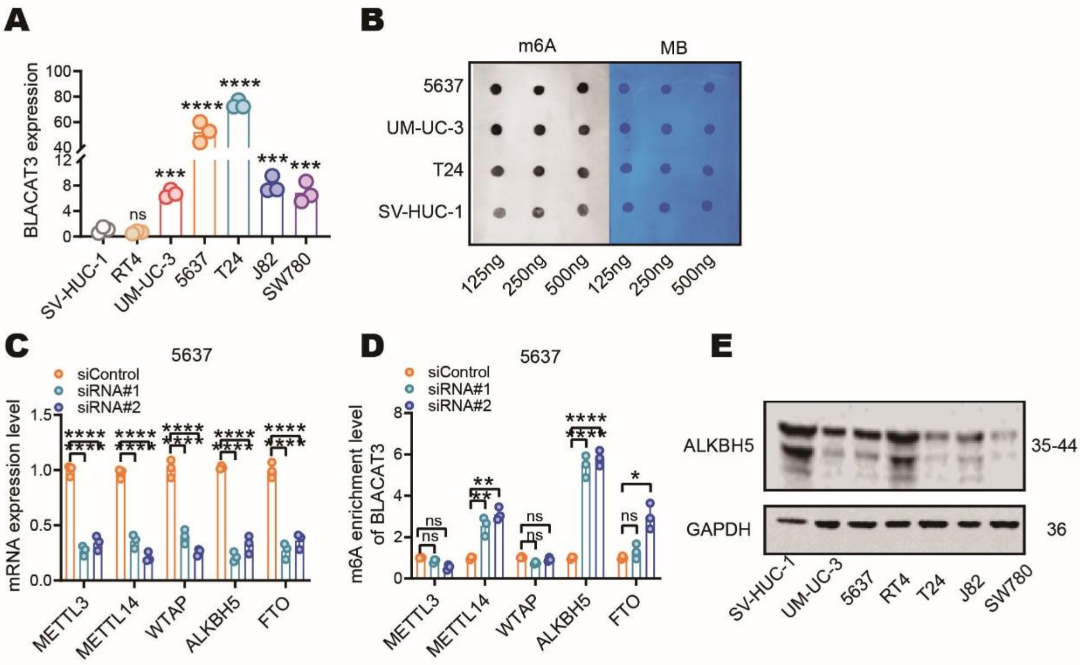

Supplementary Figure 2. A. QRT-PCR assay was performed to determine BLACAT3 expression in different BLCa cell lines (RT4, UM-UC-3, 5637, T24, J82 and SW780) and normal urothelial cell line (SV-HUC-1). **B.** Anti-m6A dot blot assay was performed to detect the overall m6A modification level of three BLCa cell lines (5637, UM-UC-3 and T24) and normal urothelial cell line (SV-HUC-1). **C.** QRT-PCR assay was used to verify the knockdown efficiency of three main RNA methyltransferases (METTL3, METTL14 and WTAP) and two typical RNA demethylases (ALKBH5 and FTO) in 5637 cells. **D.** MeRIP and qRT-PCR assay were performed to detect BLACAT3 m6A modification proportion after knockdown of five m6A regulatory enzymes in 5637 cells. **E.** WB assay was conducted to investigate the protein level of ALKBH5 in different cell lines. Statistical significance was assessed using two-tailed Student's t test between two groups, *$P < 0.05$, **$P < 0.01$, ***$P < 0.001$, ****$P < 0.0001$. Abbreviations: ns, no significance; qRT-PCR, Quantitative Real-Time Polymerase Chain Reaction; MeRIP, Methylated RNA Immunoprecipitation; WB, Western blots.

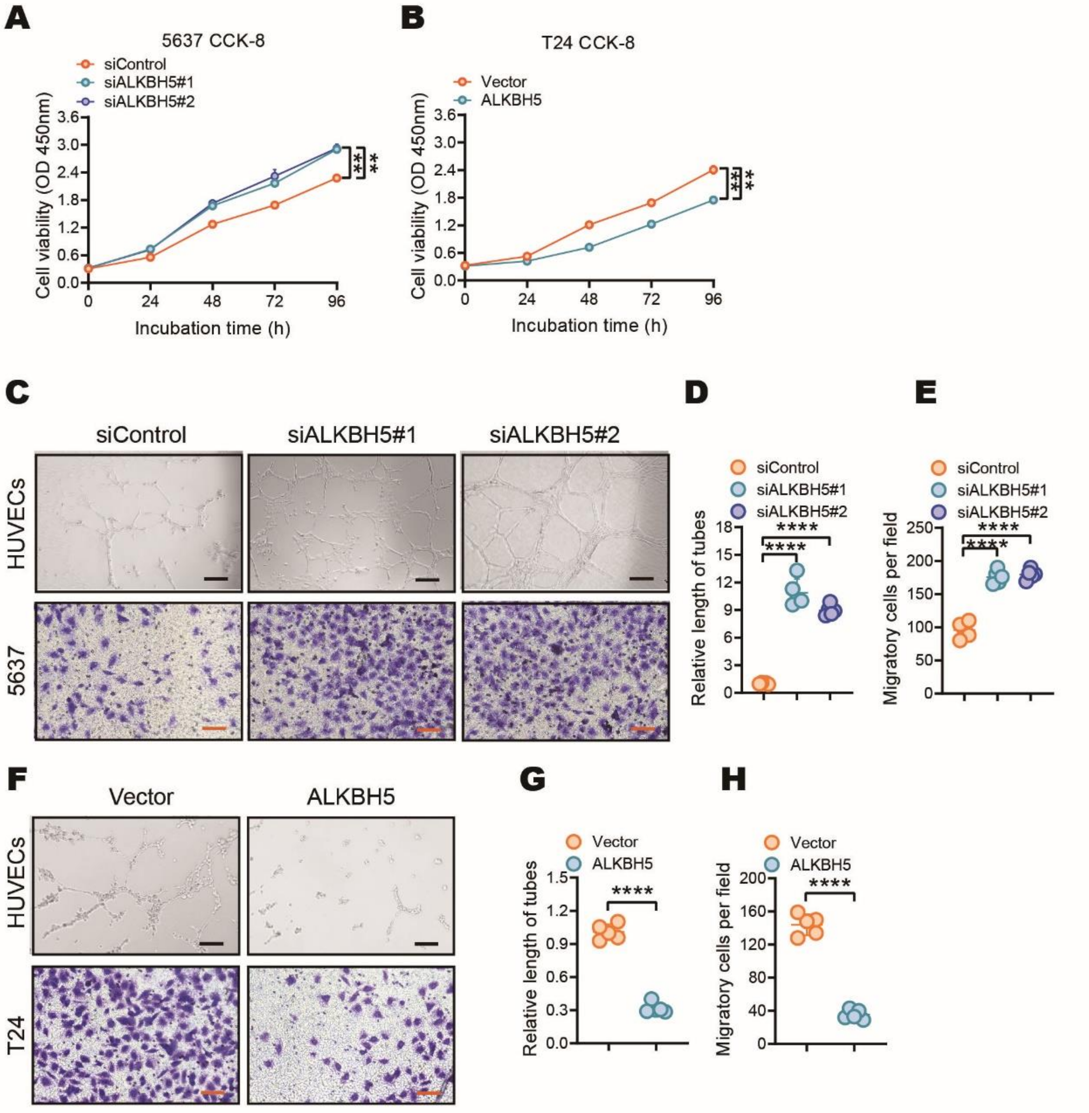

Supplementary Figure 3. The effects of ALKBH5 on BLCa angiogenesis and migration in vitro. A-B. CCK-8 assay was conducted to determine the regulatory effect of ALKBH5 on the proliferation of 5637 and T24 cells. **C-H** HUVEC tube formation assay and transwell migration assay were performed to detect the effects of ALKBH5 knockdown (**C-E**) and overexpression (**F-H**) on the pro-angiogenic and migration of BLCa cells. Scale bars: 2mm (black lines), 500μm (orange lines). Statistical significance was assessed using two-tailed Student's t test between two groups, $^{**}P < 0.01$, $^{****}P < 0.0001$. Abbreviations: CCK-8, Cell Counting Kit-8.

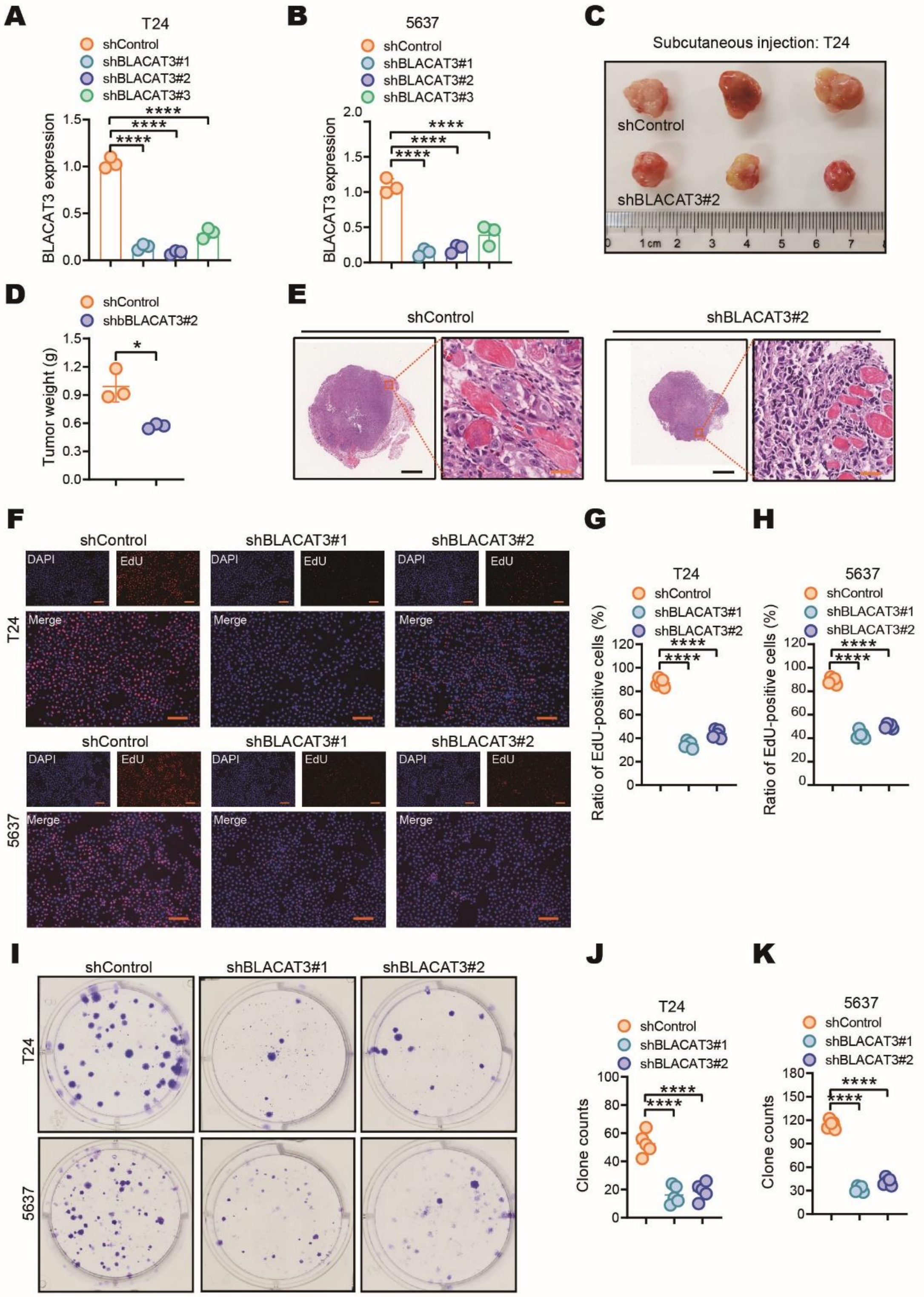

Supplementary Figure 4. Effects of BLACAT3 knockdown on proliferation of BLCa cells. A-B. QRT-PCR assays were conducted to verify the knockdown efficiency of BLACAT3 in T24 and 5637 cells. **C and D.** NSG mice were sacrificed on research endpoint (day 35) after subcutaneous injection of stably BLACAT3 knockdown T24 cells, and the subcutaneous tumors were photographed and weighed. **E.** The subcutaneous tumor was fixed and embedded and then stained with H&E. Scale bars: 1.2mm (black lines), 30 μm (orange lines). **F-H.** EdU assay was used to determine the effect of BLACAT3 knockdown on the proliferation of T24 and 5637 cells. Scale bars: 30 μm. **I-K.** Plate cloning assay was performed to detect the effect of BLACAT3 knockdown on sphere formation in T24 and 5637 cells. Statistical significance was assessed using two-tailed Student's t test between two groups, **P* < 0.05, *****P* < 0.0001. Abbreviations: qRT-PCR, Quantitative Real-Time Polymerase Chain Reaction; NSG, NOD-SCID IL-2receptor Gamma null; H&E, Haematoxylin and Eosin; EdU, 5-Ethynyl-2'-deoxyuridine.

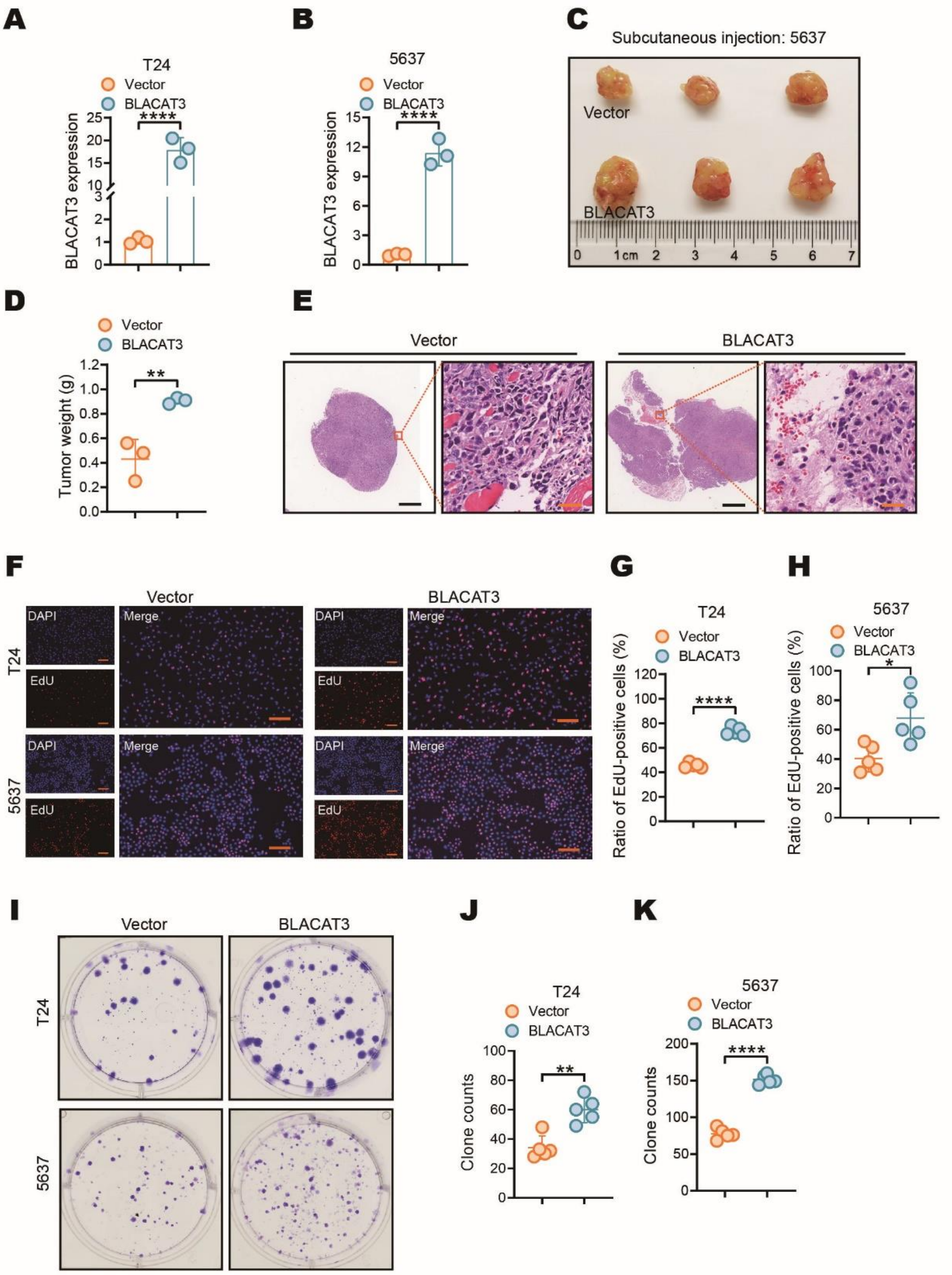

Supplementary Figure 5. Effects of BLACAT3 overexpression on proliferation of BLCa cells. A-B. QRT-PCR assay was used to verify the overexpression efficiency of BLACAT3 in T24 and 5637 cells. **C-D.** NSG mice were sacrificed on the research endpoint (day 35) after subcutaneous injection of 5637 cells stably BLACAT3 overexpression, and the subcutaneous tumors were photographed and weighed. **E.** The subcutaneous tumor was fixed, embedded and then stained with H&E. Scale bars: 1.2mm (black lines), 30 μm (orange lines). **F-H.** EdU assay was conducted to detect the effect of BLACAT3 overexpression on the proliferation of T24 and 5637 cells. Scale bars: 30 μm. **I-K.** Plate cloning assay was performed to examine the effect of BLACAT3 overexpression on sphere formation in T24 and 5637 cells. Statistical significance was assessed using two-tailed Student's t test between two groups, **P* < 0.05, ***P* < 0.01, *****P* < 0.0001. Abbreviations: qRT-PCR, Quantitative Real-Time Polymerase Chain Reaction; NSG, NOD-SCID IL-2receptor Gamma null; H&E, Haematoxylin and Eosin; EdU, 5-Ethynyl-2'-deoxyuridine.

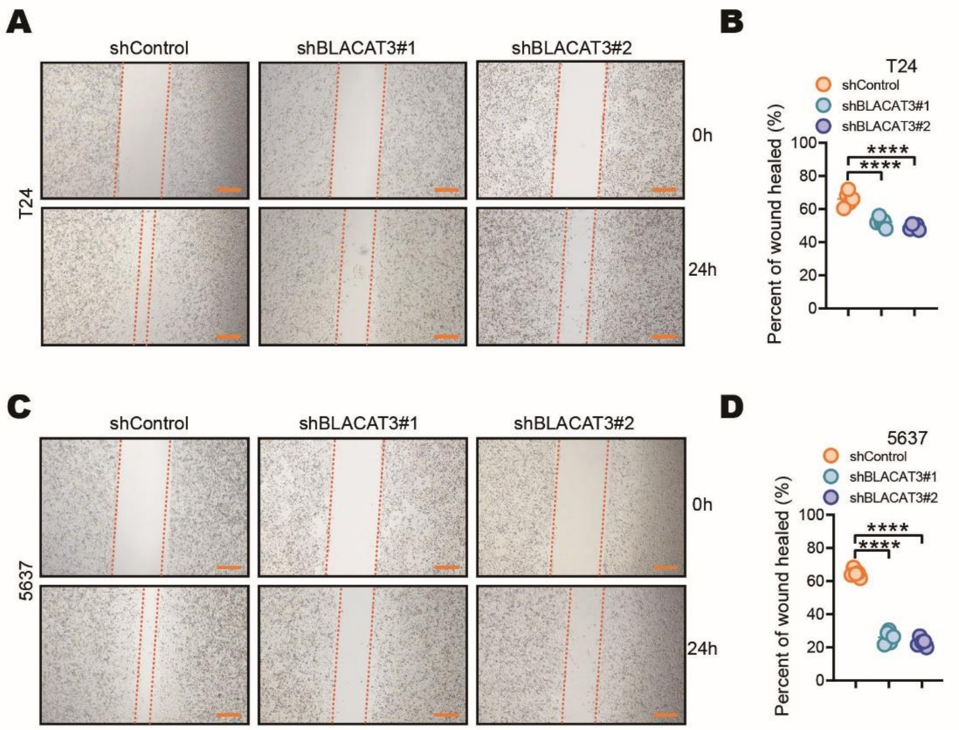

Supplementary Figure 6. Effects of BLACAT3 knockdown on the migration ability of BLCa cells. A-D. Wound healing assay was used to detect the effect of BLACA3 knockdown on the migration of T24 and 5637 cells. Scale bars: 5 mm. Statistical significance was assessed using two-tailed Student's t test between two groups, ****$P < 0.0001$.

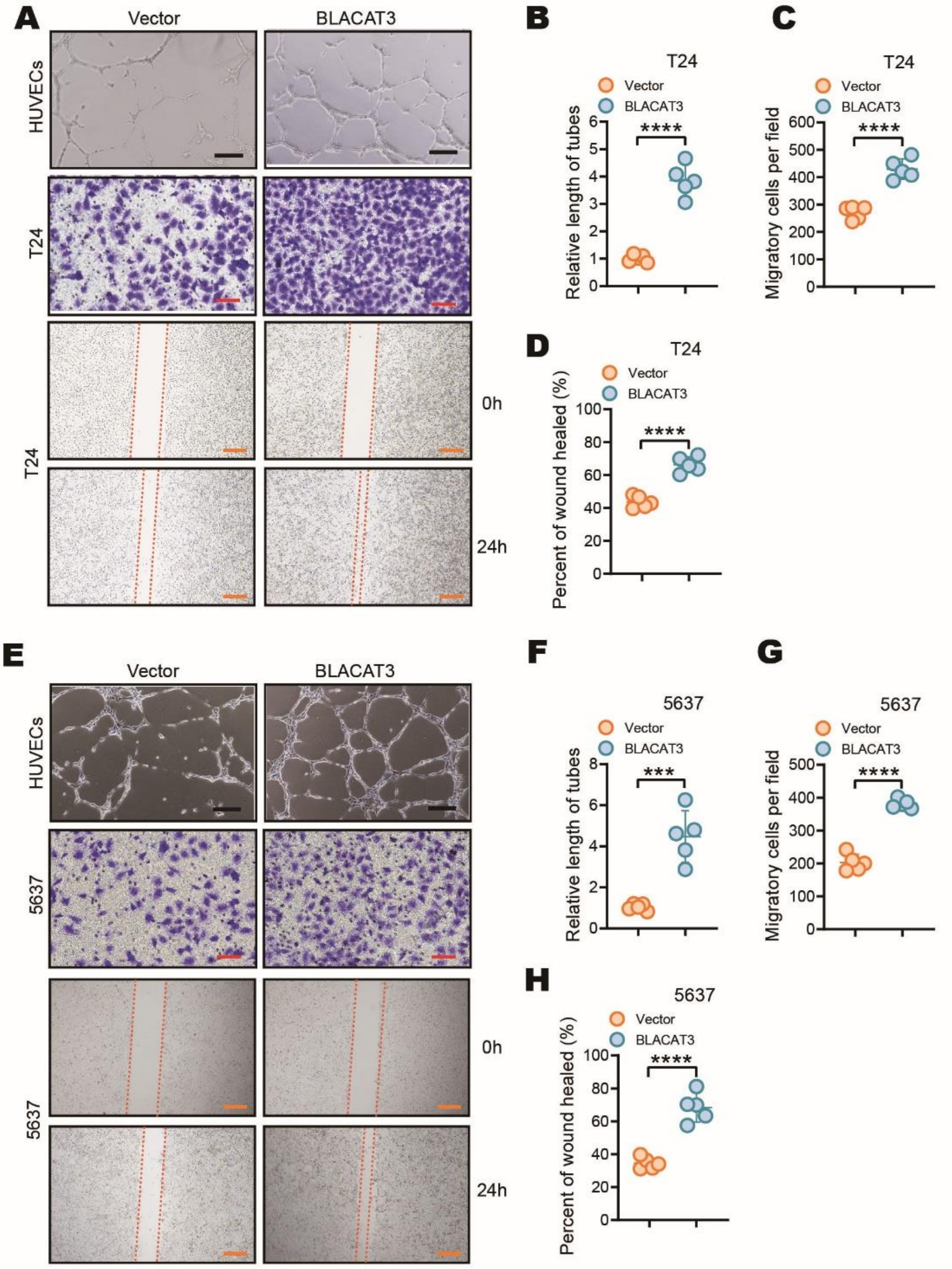

Supplementary Figure 7. Effects of BLACAT3 overexpression on angiogenesis and migration of BLCa cells in vitro. A-H. HUVEC tube formation assay, transwell migration assay and wound healing assay were performed to examine the effect of BLACAT3 overexpression on the angiogenesis and migration of T24 (**A-D**) and 5637 (**E-H**) cells. Scale bars: 2 mm (black lines), 500 μm (red lines), 5 mm (orange lines). Statistical significance was assessed using two-tailed Student's t test between two groups, $^{***}P < 0.001$, $^{****}P < 0.0001$. Abbreviations: HUVEC, Human Umbilical Vein Endothelial Cell.

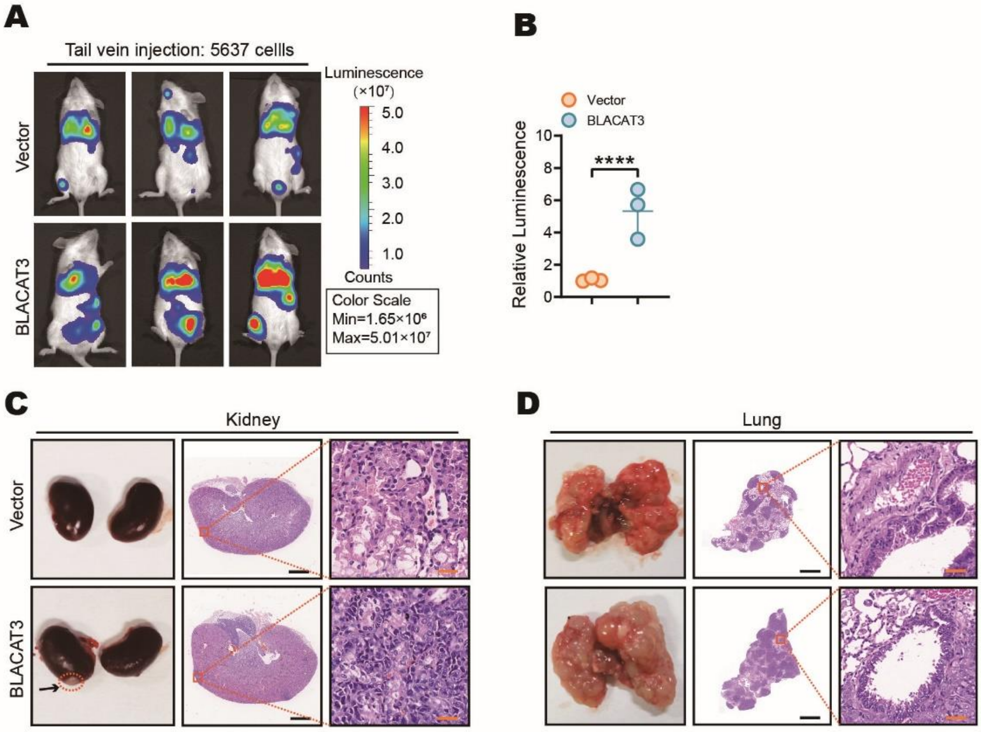

Supplementary Figure 8. Effects of BLACAT3 overexpression on BLCa cell hematogenous metastasis in vivo. A and B. In vivo imaging was performed on day 28 after tail vein injection of 5637 cells with stable BLACAT3 overexpression into NSG mice, and fluorescence quantitative statistics were performed on representative images. **C and D.** NSG mice were sacrificed on the 28th day after tail vein injection model was constructed, and gross samples of kidney and lung were isolated, fixed, embedded and sliced for H&E staining. Scale bars: 1.2 mm (black lines), 30 μm (orange lines). Statistical significance was assessed using two-tailed Student's t test between two groups, ****$P < 0.0001$. Abbreviations: NSG, NOD-SCID IL-2receptor Gamma null; H&E, Haematoxylin and Eosin.

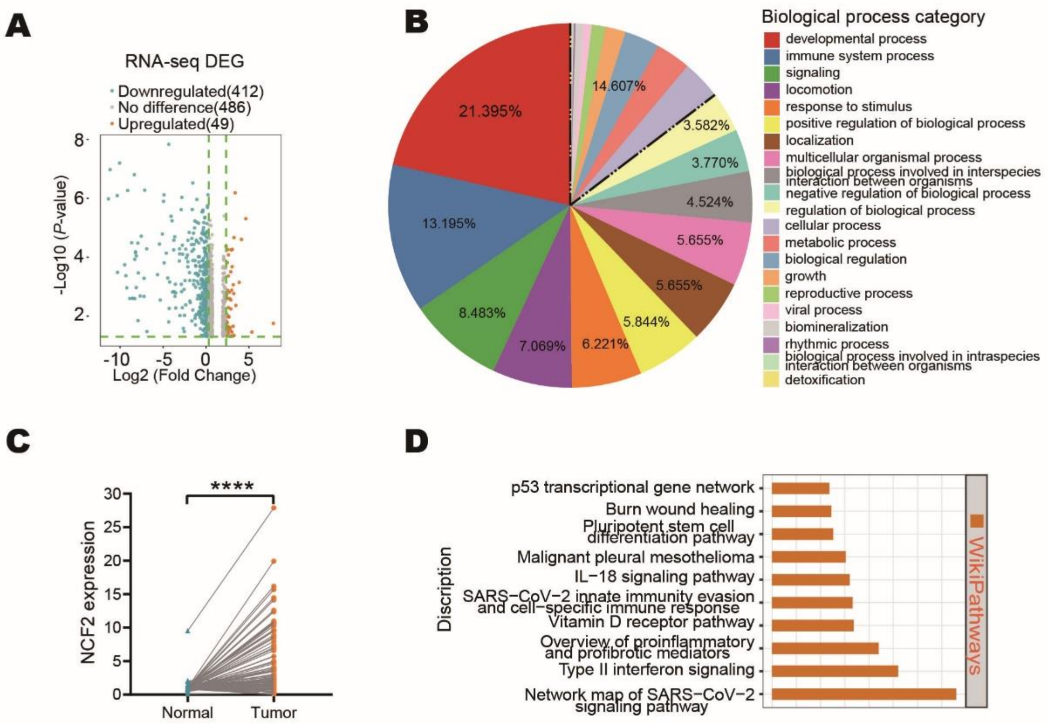

Supplementary Figure 9. RNA-sequencing analysis. A. Volcano plot showed the proportion of differentially expressed genes after BLACAT3 knockdown. **B.** GO analysis of biological functions enriched by differentially expressed genes after BLACAT3 knockdown. **C.** QRT-PCR assay was performed to detect NCF2 expression in clinical BLCa tumor tissues and adjacent normal tissues (n = 104). **D.** WikiPathways analysis of signaling pathways enriched based on differentially expressed genes after BLACAT3 knockdown. Statistical significance was assessed using two-tailed Student's t test between two groups, ****$P < 0.0001$. Abbreviations: GO, Gene Ontology; qRT-PCR, Quantitative Real-Time Polymerase Chain Reaction.

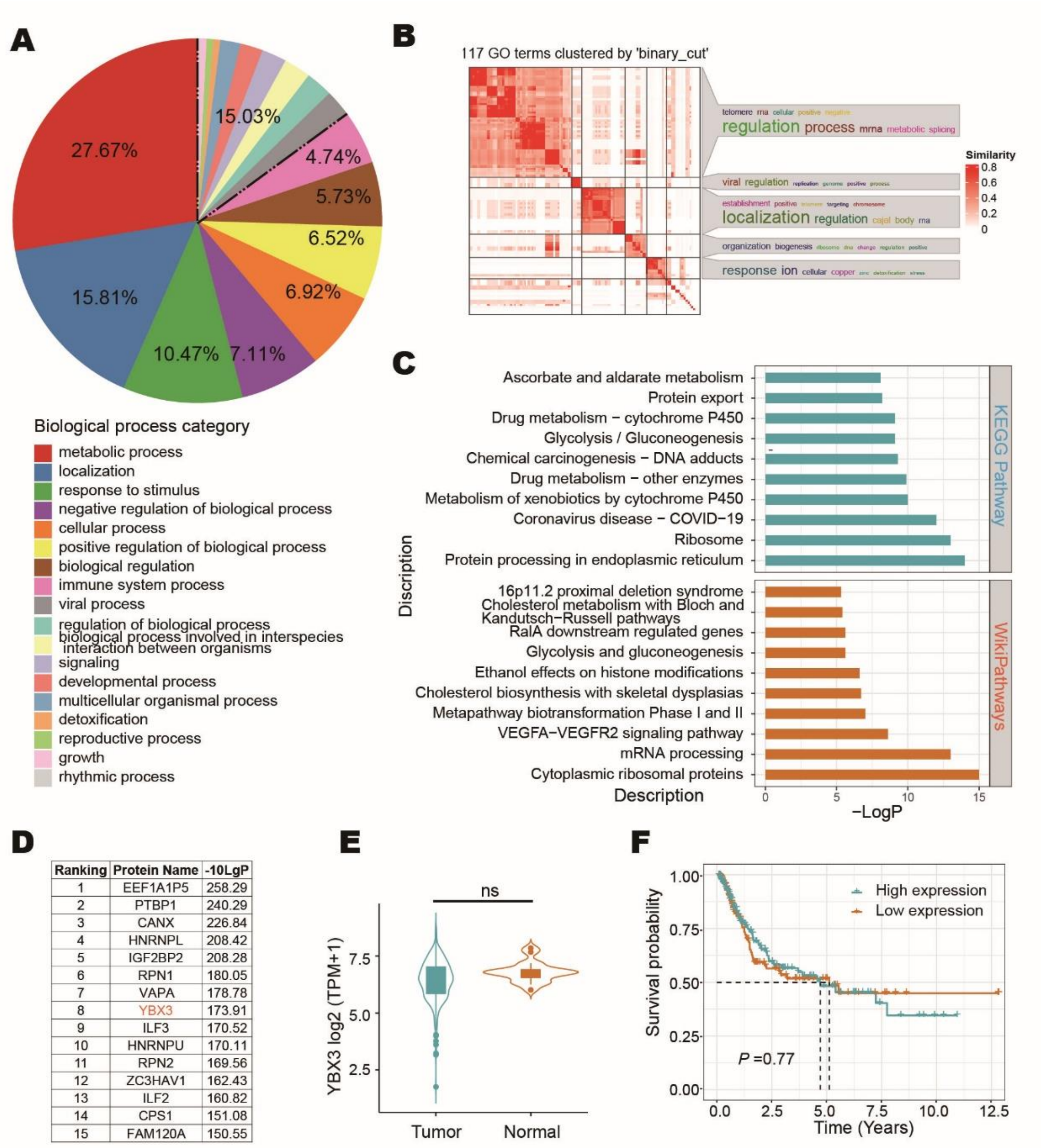

Ranking	Protein Name	-10LgP
1	EEF1A1P5	258.29
2	PTBP1	240.29
3	CANX	226.84
4	HNRNPL	208.42
5	IGF2BP2	208.28
6	RPN1	180.05
7	VAPA	178.78
8	YBX3	173.91
9	ILF3	170.52
10	HNRNPU	170.11
11	RPN2	169.56
12	ZC3HAV1	162.43
13	ILF2	160.82
14	CPS1	151.08
15	FAM120A	150.55

Supplementary Figure 10. YBX3 identification by MS and functional enrichment analysis. A and B. GO analysis of biological functions enriched by BLACAT3-binding proteins identified by MS. **C.** KEGG and WikiPathways analysis of signaling pathways enriched by BLACAT3-binding proteins. **D.** The top 15 RNA Binding proteins pulled down by BLACAT3. **E.** Differential expression profile of mRNAs between 411 BLCa tumor tissues and 19 normal bladder tissues in the TCGA-BLCa dataset showed that YBX3 was not differentially expressed. **F.** The relationship between the mRNA level of YBX3 and BLCa patient OS was analyzed based on the TCGA data ($P > 0.05$). Abbreviations: ns, no significance; GO, Gene Ontology; KEGG, Kyoto Encyclopedia of Genes and Genomes; TCGA, The Cancer Genome Atlas; OS, Overall Survival.

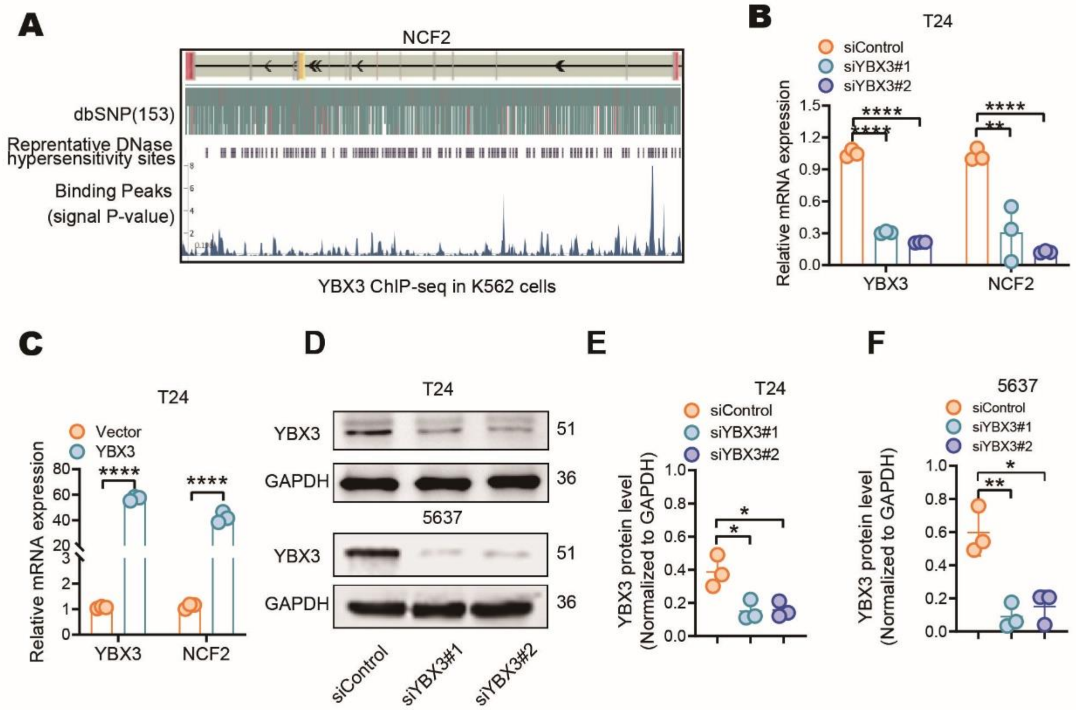

Supplementary Figure 11. YBX3 binds to NCF2 promoter and regulate transcription. A. ChIP-sequencing data (https://www.encodeproject.org/) in K562 cells showed the sequence site of YBX3 binding to NCF2. **B.** QRT-PCR assay was performed to verify the knockdown efficiency of YBX3, and detected the effect of YBX3 knockdown on NCF2 mRNA expression in T24 cells. **C.** QRT-PCR assays were performed to verify the overexpression efficiency of YBX3, and detect the effect of YBX3 overexpression on NCF2 mRNA expression in T24 cells. **D-F.** WB verified the knockdown efficiency of YBX3 in T24 and 5637 cell lines. Statistical significance was assessed using two-tailed Student's t test between two groups, **P* < 0.05, ***P* < 0.01, *****P* < 0.0001. Abbreviations: ChIP, Chromatin immunoprecipitation; qRT-PCR, Quantitative Real-Time Polymerase Chain Reaction.

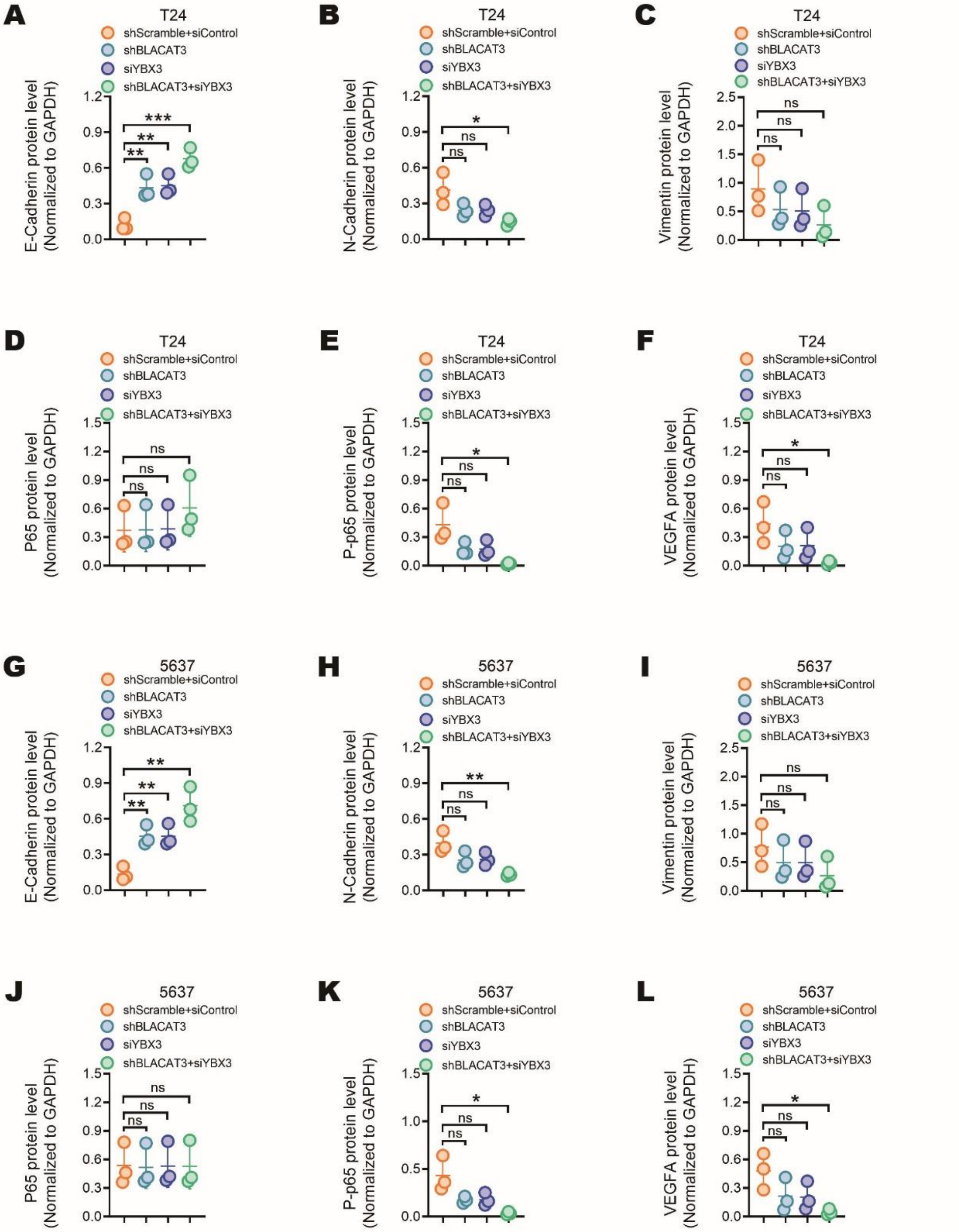

Supplementary Figure 12. Statistical histogram of WB bands. Statistical significance was assessed using two-tailed Student's t test between two groups. **P* < 0.05, ***P* < 0.01, ****P* < 0.001. Abbreviation: ns, no significance.

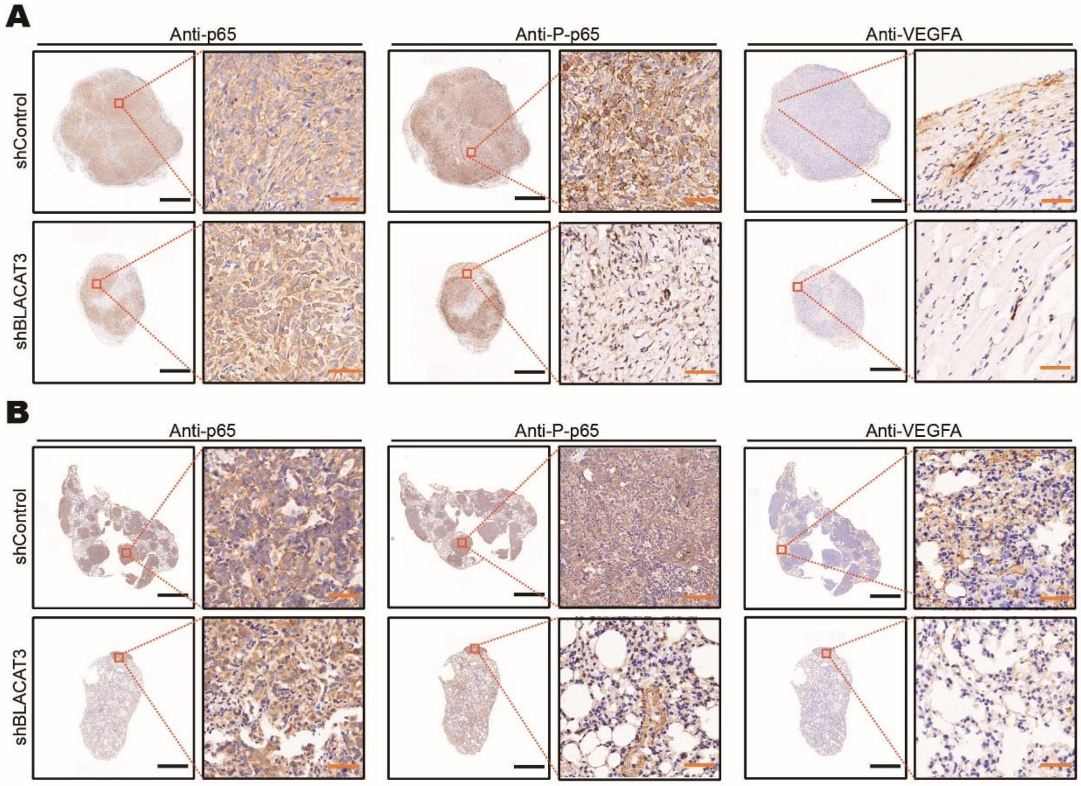

Supplementary Figure 13. BLACAT3 promotes BLCa proliferation and metastasis via acivating NF-κB signaling pathway. A. IHC asaay was conducted to investigate the effect of BLACAT3 on NF-κB signaling pathway in subcutaneous xenograft model. **B.** IHC asaay was conducted to investigate the effect of BLACAT3 on NF-κB signaling pathway in hematogenous metastasis model. Abbreviation: IHC, immunohistochemistry.

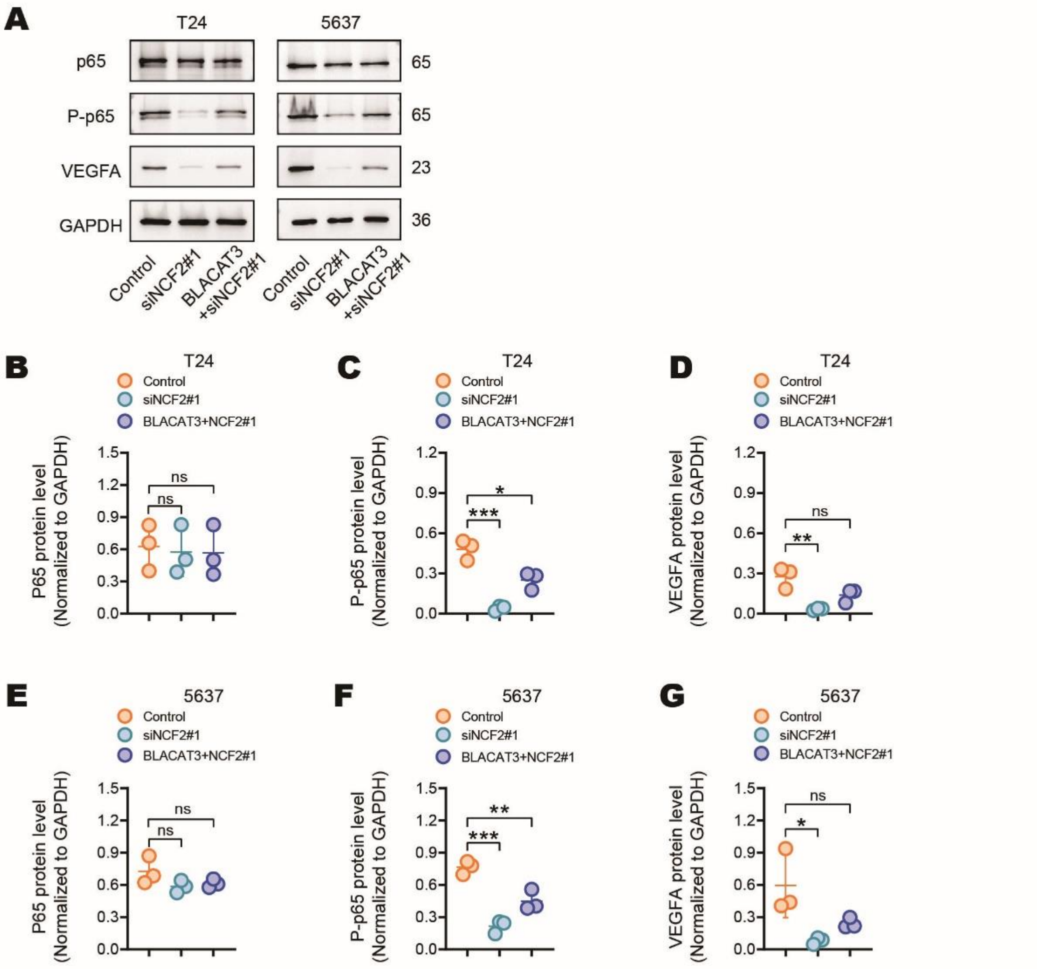

Supplementary Figure 14. BLACAT3/NCF2 axis can activate NF-κB signaling pathway. A. WB assay was conducted to investigate the effect of BLACAT3/NCF2 axis on NF-κB signaling pathway. **B.** Statistical histogram of WB bands. Statistical significance was assessed using two-tailed Student's t test between two groups. **P* < 0.05, ***P* < 0.01, ****P* < 0.001. Abbreviation: ns, no significance.

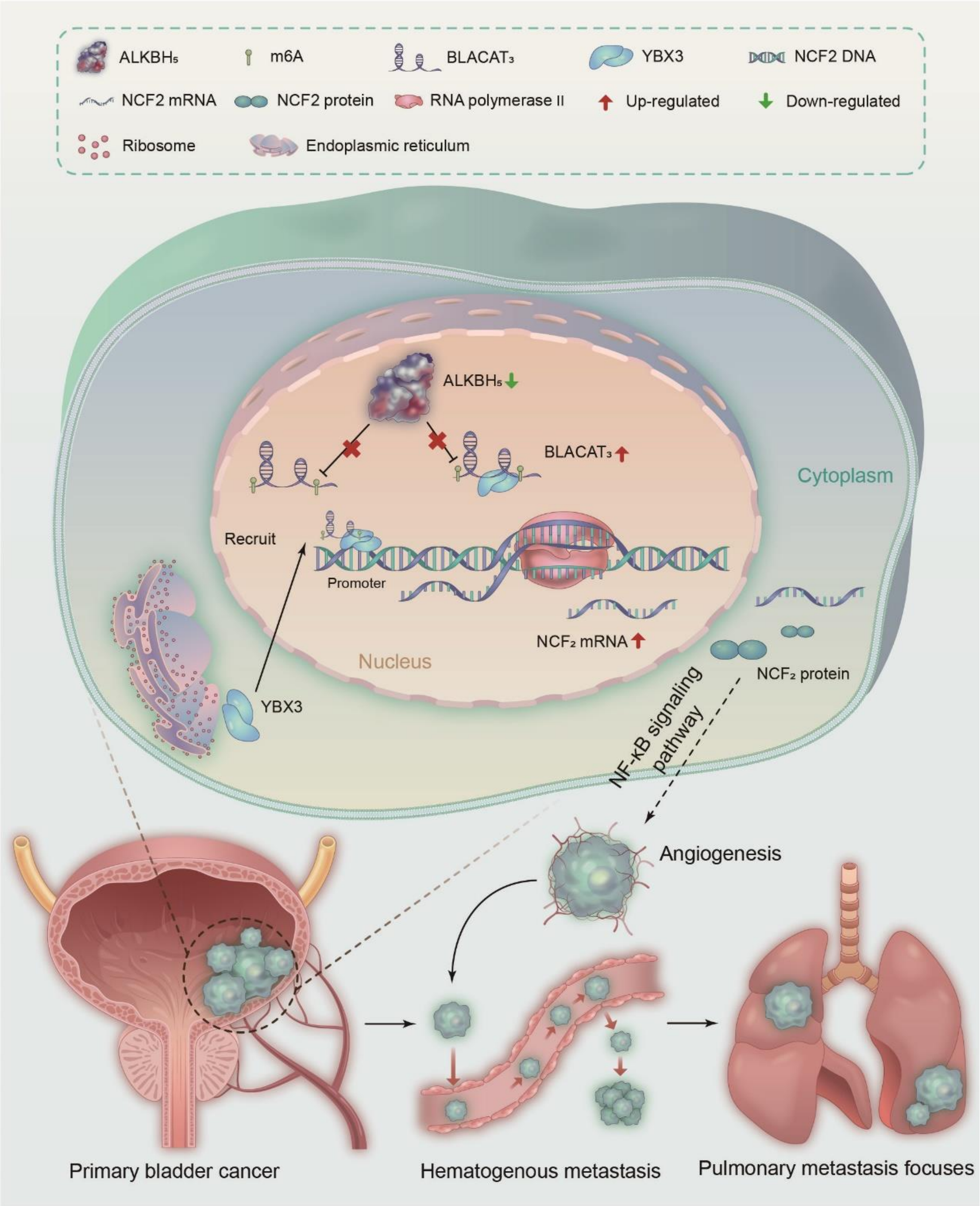

Supplementary Figure 15. Mechanism diagram by which BLACAT3 promotes BLCa tumor angiogenesis and metastasis. M6A modification contributes to BLACAT3 upregulation by stabilizing RNA structure. BLACAT3 induces YBX3 to shuttle into the nucleus, synergistically enhances NCF2 transcription, and promotes BLCa angiogenesis and metastasis via activating NF-κB signaling pathway.
